# Gut *Bifidobacterium pseudocatenulatum* protects against fat deposition by enhancing secondary bile acid biosynthesis

**DOI:** 10.1002/imt2.261

**Published:** 2024-12-30

**Authors:** Andong Zha, Ming Qi, Yuankun Deng, Hao Li, Nan Wang, Chengming Wang, Simeng Liao, Dan Wan, Xia Xiong, Peng Liao, Jing Wang, Yulong Yin, Bi'e Tan

**Affiliations:** ^1^ Key Laboratory of Hunan Province for the Products Quality Regulation of Livestock and Poultry College of Animal Science and Technology, Hunan Agricultural University Changsha China; ^2^ Yuelushan Laboratory Hunan China; ^3^ School of Basic Medical Science, Central South University Changsha China; ^4^ Laboratory of Animal Nutritional Physiology and Metabolic Process, Key Laboratory of Agro‐Ecological Processes in Subtropical Region, National Engineering Laboratory for Pollution Control and Waste Utilization in Livestock and Poultry Production, Institute of Subtropical Agriculture, Chinese Academy of Sciences Changsha China

**Keywords:** *Bifidobacterium pseudocatenulatum*, bile salt hydrolase, fat deposition, lithocholic acid, secondary bile acid biosynthesis

## Abstract

Gut microbiome is crucial for lipid metabolism in humans and animals. However, how specific gut microbiota and their associated metabolites impact fat deposition remains unclear. In this study, we demonstrated that the colonic microbiome of lean and obese pigs differentially contributes to fat deposition, as evidenced by colonic microbiota transplantation experiments. Notably, the higher abundance of *Bifidobacterium pseudocatenulatum* was significantly associated with lower backfat thickness in lean pigs. Microbial‐derived lithocholic acid (LCA) species were also significantly enriched in lean pigs and positively correlated with the abundance of *B. pseudocatenulatum*. In a high‐fat diet (HFD)‐fed mice model, administration of live *B. pseudocatenulatum* decreased fat deposition and enhances colonic secondary bile acid biosynthesis. Importantly, pharmacological inhibition of the bile salt hydrolase (BSH), which mediates secondary bile acid biosynthesis, impaired the anti‐fat deposition effect of *B. pseudocatenulatum* in antibiotic‐pretreated, HFD‐fed mice. Furthermore, dietary LCA also decreased fat deposition in HFD‐fed rats and obese pig models. These findings provide mechanistic insights into the anti‐fat deposition role of *B. pseudocatenulatum* and identify BSH as a potential target for preventing excessive fat deposition in humans and animals.

## Highlights

- The gut microbiota composition and function differ significantly between lean and obese pigs, and breed‐specific microbiomes regulate fat deposition.
- Gut *Bifidobacterium pseudocatenulatum* protects against fat deposition by enhancing secondary bile acid biosynthesis.
- Dietary supplementation with lithocholic acid also helps prevent fat deposition.

## Graphical abstract

This figure highlights how gut microbiota and bile acids regulate fat deposition in lean and obese pigs. Lean pigs showed increased microbial‐derived lithocholic acid (LCA) species and muricholic acid species, closely linked to the abundance of *Bifidobacterium pseudocatenulatum*. In contrast, obese pigs exhibited gut microbiota favoring hyodeoxycholic acid species accumulation. In high‐fat diet‐fed mice, live *B. pseudocatenulatum* supplementation enhances secondary bile acid synthesis, especially LCA production, and reduces fat mass, while bile salt hydrolase (BSH) inhibition negates these effects. Dietary LCA similarly lowers fat deposition in obese pigs and rats. These findings provide mechanistic insights into the anti‐fat deposition role of *B. pseudocatenulatum* and identify BSH as a potential target for preventing excessive fat deposition in humans and animals.

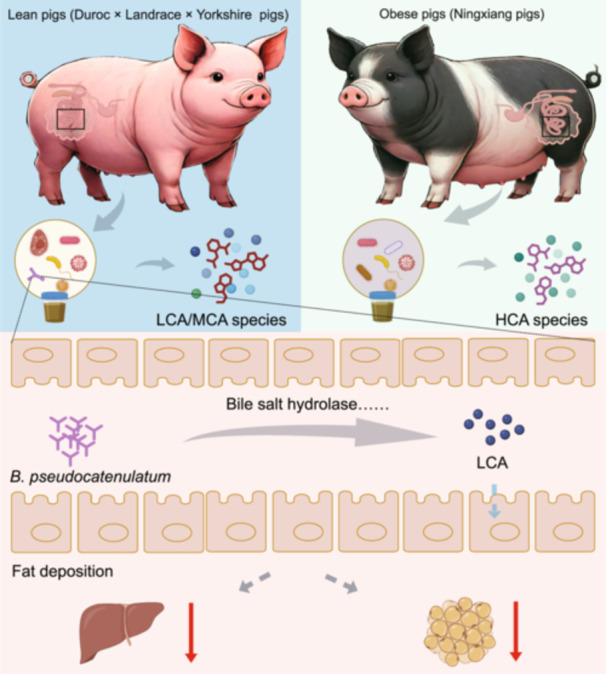

## INTRODUCTION

Excessive fat deposition is prone to developing metabolic syndrome including obesity, type II diabetes mellitus, nonalcoholic fatty liver disease, and cardiovascular disease in humans [1]. It can also negatively affect the feed conversion and production efficiency in livestock production [2]. Recent studies have highlighted the critical role of gut microbiota in energy homeostasis and fat deposition [2, 3, 4]. Therefore, targeting specific microbial species and their associated metabolites may present a promising strategy for reducing fat deposition.

The relative abundance of gut microbiota varies significantly in response to factors, such as diet, geographic location, and physiological state [5]. Notably, the abundance of *Bifidobacterium* spp., which plays an important role in energy homeostasis, is significantly decreased in obese individuals compared with healthy individuals [6]. Colonization with *Bifidobacterium pseudocatenulatum* strain C95 has been shown to reduce body weight, fat mass, fasting blood glucose, and insulin resistance and improve postprandial blood glucose response. Furthermore, it increased cecal acetic acid content in high‐fat diet (HFD)‐fed mice. In addition, inoculation with the *B. pseudocatenulatum* strain also lowered fasting blood glucose in germ‐free mice colonized with fecal microbiota from type II diabetes patients through modulation of metabolites, including bile acids (BAs) [7]. However, the mechanistic understanding of how *Bifidobacterium* spp. influences host fat deposition remains significantly underexplored. Several studies have suggested pivotal roles for BAs produced by commensal bacteria in regulating host energy homeostasis [8, 9]. For example, hyocholic acid (HCA) species have been identified as a biomarker for metabolic disorders, and microbial‐derived hyodeoxycholic acid (HDCA) has been shown to alleviate nonalcoholic fatty liver disease via farnesoid X receptor (*Fxr*) and peroxisome proliferator‐activated receptors α (*Pparα*) [10, 11, 12]. Furthermore, *Bifidobacterium* spp. is implicated in secondary bile acids (BA) metabolism [13], suggesting that secondary BAs may play a crucial role in *Bifidobacterium*‐mediated effects. Despite these findings, there is a notable lack of literature exploring this hypothesis in depth, leaving an important aspect of microbial‐host interactions underexplored.

The digestive physiology and omnivorous feeding behavior of pigs are similar to those of humans. Therefore, local obese pig species such as Ningxiang (NX) pig, known for its thick backfat, serve as ideal models for studying fat deposition and obesity [14, 15, 16]. In this study, integrating shotgun sequencing and colonic metabolome was utilized to comprehensively characterize the difference in microbial community composition and metabolite profiles between lean and obese pigs. We aimed to identify key strains and key pathways that affected fat deposition and provide a potential treatment strategy for decreasing fat deposition in humans and animals.

## RESULTS

### Distinct microbial profile associated with backfat deposition in lean and obese pigs

**Figure 1:**
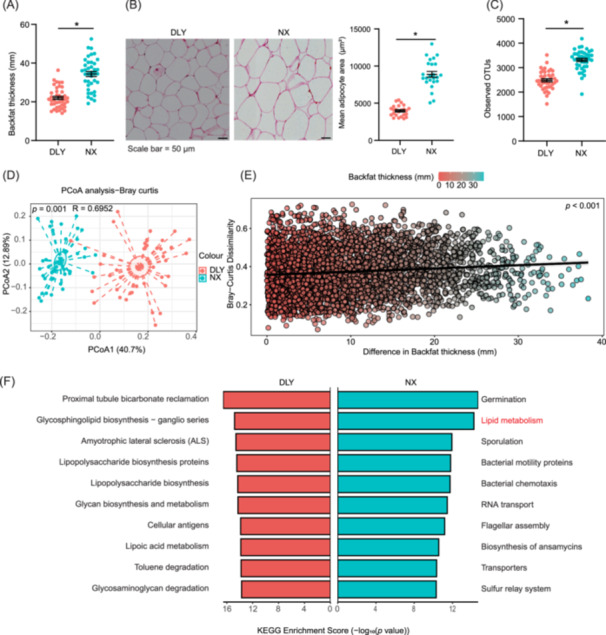
Distinct microbial profile associated with backfat deposition in lean and obese pigs. (A) Backfat thickness. Data are shown as mean ± SEMs. *n* = 40 per group, *p < 0.05 (Student's *t* test). (B) Representative hematoxylin and eosin staining images of subcutaneous adipose tissue (100×). Data are shown as mean ± SEMs. *n* = 40 per group, *p < 0.05 (Student's *t* test). (C) Colonic microbial *α* diversity based on observed operational taxonomic units. Data are shown as mean ± SEMs, *n* = 40 per group, *p < 0.05 (Student's *t* test). (D) Colonic microbial principal coordinates analysis based on Bray–Curtis distances. (E) The association between gut microbiome and backfat thickness (Mantel test). (F) Top 20 differential pathways based on phylogenetic investigation of communities by reconstruction of unobserved states 2.0 analysis. DLY group, Duroc × Landrace × Yorkshire pigs; NX group, Ningxiang pigs; SEM, standard error of the mean.

Compared to lean pigs (Duroc × Landrace × Yorkshire (DLY) pigs), backfat thickness and adipocyte size in subcutaneous adipose tissue (SAT) were significantly greater in NX pigs (Figure 1A,B). The hindgut microbiota is crucial for energy homeostasis and fat deposition, primarily enriched in the colon [4]. Colonic digesta was collected for 16S rRNA sequencing, revealing that the diversity and richness of colonic microbiota in NX pigs were statistically higher than DLY pigs (Figure 1C). Principal coordinates analysis (PCoA) also revealed significant differences in the colonic microbial community structure between DLY and NX pigs (Figure 1D). To discern the roles of colonic microbiome in fat deposition, we correlated community composition dissimilarities with backfat thickness using the mantel test, which indicated a significant correlation (*r* = 0.1492, *p *< 0.001, Figure 1E). Phylogenetic investigation of communities by reconstruction of unobserved states 2 analysis showed that the lipid metabolism pathway was significantly enriched in the colonic microbiota of NX pigs (Figure 1F). Moreover, pathways associated with secondary BA biosynthesis, primary BA biosynthesis, glycerolipid metabolism, biosynthesis of unsaturated fatty acids, synthesis and degradation of ketone bodies, glycerophospholipid metabolism, and linoleic acid metabolism were also enriched in NX pig (Figure S1). These results indicated that the colonic microbial profile may regulate fat deposition in pigs.

### The gut microbiome of lean and obese pigs contributes to the variations of lipid deposition

**Figure 2:**
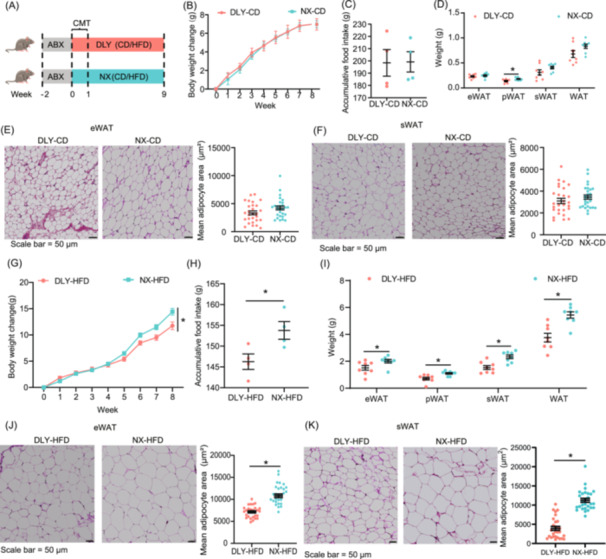
The gut microbiome of lean and obese pigs contributes to the variations of lipid deposition. (A) Schematic of the colonic microbiota transplantation experiment. C57BL/6 male mice pretreated with antibiotic cocktail for 2 weeks, and received colonic microbiota suspension of Ningxiang (NX) or Duroc × Landrace × Yorkshire (DLY) pigs for 1 week. Mice were fed with chow–diet (CD)/high‐fat diet (HFD) for 9 weeks. (B) Body weight change; DLY‐CD: Mice received colonic microbiome from DLY pigs and fed with chow diet; NX‐CD: Mice received colonic microbiome from NX pigs and fed with chow diet. Data are shown as mean ± SEMs, *n* = 8 per group, *p < 0.05 (repeated measures analysis of variance). (C) Accumulative food intake. Data are shown as mean ± SEMs, *n* = 4 per group, *p < 0.05 (Student's *t* test). (D) Organ weight; epididymal white adipose tissue (eWAT); perirenal white adipose tissue (pWAT); subcutaneous white adipose tissue (sWAT); white adipose tissue (WAT). Data are shown as mean ± SEMs, *n* = 8 per group, *p < 0.05 (Student's *t* test). (E) Representative hematoxylin and eosin (H&E)‐stained image of eWAT, and quantification of the mean adipocytes area. Data are shown as mean ± SEMs, *n* = 4 per group, *p < 0.05 (Student's *t* test), Scale bar = 50 μm. (F) Representative H&E‐stained image of sWAT and quantification of the mean adipocytes area. Data are shown as mean ± SEMs, *n* = 4 per group, *p < 0.05 (Student's *t* test), Scale bar = 50 μm. (G) Body weight change; DLY‐HFD: Mice received colonic microbiome from DLY pigs and fed with HFD; NX‐CD: Mice received colonic microbiome from NX pigs and fed with HFD. Data are shown as mean ± SEMs, *n* = 8 per group, *p < 0.05 (repeated measures analysis of variance). (H) Accumulative food intake. Data are shown as mean ± SEMs, *n* = 4 per group, *p < 0.05 (Student's *t* test). (I) Adipose tissue weight. epididymal white adipose tissue (eWAT), perirenal white adipose tissue (pWAT), subcutaneous white adipose tissue (sWAT), white adipose tissue (WAT). Data are shown as mean ± SEMs, *n* = 8 per group, *p < 0.05 (Student's *t* test). (J) Representative H&E‐stained image of eWAT, and quantification of the mean adipocytes area. Data are shown as mean ± SEMs, *n* = 4 per group, *p < 0.05 (Student's *t* test), Scale bar = 50 μm. (K) Representative H&E‐stained image of sWAT, and quantification of the mean adipocytes area. Data are shown as mean ± SEMs, *n* = 4 per group, * *p* < 0.05 (Student's *t* test), Scale bar = 50 μm. DLY‐CD: Mice received colonic microbiome from DLY pigs and fed with chow diet; NX‐CD: Mice received colonic microbiome from NX pigs and fed with chow diet; DLY‐HFD: Mice received colonic microbiome from DLY pigs and fed with HFD; NX‐CD: Mice received colonic microbiome from NX pigs and fed with HFD. SEM, standard error of the mean.

To investigate the impact of pig colonic microbiota on fat deposition under controlled conditions, we transplanted colonic microbiota from DLY and NX pigs to 7–8 week‐old male C57BL/6J mice (Figure 2A). Transplanted breed‐specific colonic microbiota did not influence body weight change or accumulative food intake in chow–diet‐fed mice (Figure 2B,C). Transplanted colonic microbiota from NX pigs significantly increased the weight of perirenal white adipose tissue (pWAT) in chow– diet‐fed mice (Figure 2D), while no significant effects were observed on the weight of subcutaneous white adipose tissue (sWAT) and epididymal white adipose tissue (eWAT), or the mean adipocyte area of eWAT and sWAT in chow–diet‐fed mice (Figure 2E,F). Additionally, there were no significant differences in serum lipid profiles, including total cholesterol, glucose, triglyceride, high‐density lipoprotein cholesterol (HDLC), low‐density lipoprotein cholesterol (LDLC), liver weight, and hepatic steatosis of chow–diet‐fed mice (Figure S2A–C). Interestingly, the mRNA expression of *Fxr* and Takeda G protein‐coupled receptor *5* (*Tgr5*) in the liver of mice transplanted with the NX pig's colonic microbiota was significantly reduced compared to those transplanted with the DLY pig's colonic microbiota (Figure S2D). Furthermore, in HFD‐fed mice, transplantation of NX pig's colonic microbiota resulted in significant increases in body weight and accumulative food intake (Figure 2G,H). These mice also exhibited a higher weight of sWAT, eWAT, pWAT, and total white adipose tissue (WAT), alongside a significant increase in the mean adipocyte size of eWAT and sWAT (Figure 2I–K). Serum glucose, triglyceride, total cholesterol, HDLC, LDLC, and nonesterified fatty acid (NEFA) were significantly increased in NX‐recipient mice (Figure S2E). BAs, which play an important role in lipid absorption, were notably reduced in the serum of NX‐recipient mice (Figure S2E). HFD is known to induce liver injury, and transplanted colonic microbiota from NX increased the weight of the liver and hepatic steatosis score in HFD‐fed mice (Figure S2F,G). In transcript level, the mRNA expressions of genes involved in lipogenesis, including acetyl‐CoA carboxylase (*Acc*), fatty acid synthase (*Fasn*), *Pparγ*, and Fxr, were markedly upregulated in the liver of NX‐recipient mice (Figure S2H). These findings collectively illustrate that the effect of colonic microbiota on fat deposition varies between obese and lean pigs, with NX pig's microbiota specifically enhancing fat accumulation, particularly under HFD conditions.

### Colonic‐specific microbiome mediates variations in secondary BA metabolism between lean and obese pigs

**Figure 3:**
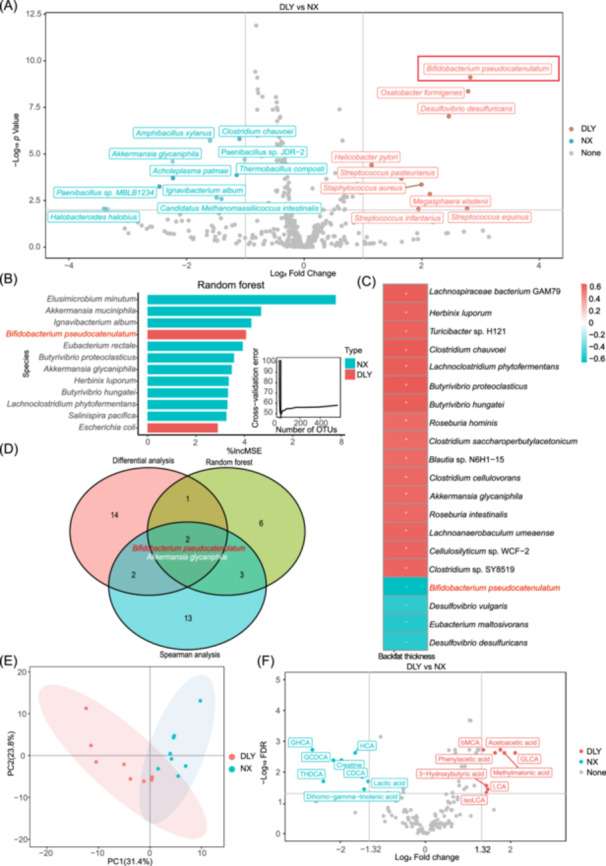
Colonic‐specific microbiome mediates variations in secondary bile acid metabolism between lean and obese pigs. (A) Volcano plot illustrating differential bacterial species between Duroc × Landrace × Yorkshire (DLY) and Ningxiang (NX) pigs (false discovery rate [FDR] < 0.01, Log_2_ fold change > 2 or Log_2_ fold change < −2), *n* = 25. None indicates species with no significant differences; DLY indicates species enriched in DLY pigs; NX indicates species enriched in NX pigs. (B) Twelve bacterial species were identified as bacterial biomarkers to predict backfat thickness by random forest analysis, *n* = 25. (C) Two‐tailed spearman correlation coefficient analysis of bacterial species, and backfat thickness (Top 20). + means positively correlation; − means negatively correlation. (D) Venn diagram of differential analysis, random forest, and Spearman analysis. (E) Principal component analysis score plot of colonic metabolomics, *n* = 8. (F) Volcano plot showing differential metabolites between DLY and NX pigs (FDR < 0.05, Log_2_ fold change > 1.32 or Log_2_ fold change < −1.32), *n* = 8. None indicates metabolites with no significant differences; DLY indicates metabolites enriched in DLY pigs; NX indicates metabolites enriched in NX pigs.

To further identify the specific gut microbes mediating backfat thickness, we performed shotgun metagenomic sequencing of 50 colonic chyme samples. We identified 559 bacterial species and 12 archaea species, with a higher number of bacterial species in the colonic microbial community of NX pigs. Nine bacterial species were markedly enriched in DLY pigs, while 10 species were more common in NX pigs (Figure 3A). Employing the randomForest package, we developed a sparse random forest model that highlighted 12 bacterial species, such as *B. pseudocatenulatum*, as significant predictors of backfat thickness, explaining 59.71% of the variance (Figure 3B). Next, spearman analyses identified the top 20 bacteria associated with backfat thickness, including *B. pseudocatenulatum* (Figure 3C). Through integrative differential, random forest, and spearman analyses, *B. pseudocatenulatum* and *Akkermansia glycaniphila* emerged as statistically significant correlates of backfat thickness and key biological markers (Figure 3D). Furthermore, linear discriminant analysis (LDA) effect size (LEfSe) analysis also revealed that *B. pseudocatenulatum* was enriched in DLY pigs (Figure S3). Taken together, these findings pinpointed *B. pseudocatenulatum* and *A. glycaniphila* as pivotal contributors to the variance in backfat thickness.

To elucidate the functional differences in the colonic microbiome between DLY and NX pigs, we employed HUMAnN2 for functional prediction. The analysis revealed significant enrichment of pathways related to fatty acid biosynthesis, secondary BA biosynthesis, and several other metabolic functions in NX pigs. In contrast, pathways such as mismatch repair, homologous recombination, and nucleotide excision repair were notably enriched in DLY pigs (Figure S4A). Subsequent metabolomic analysis identified 147 metabolites in the colonic metabolome, displaying distinct profiles between NX and DLY pigs (Figure 3E). Sixteen metabolites were significantly different between DLY and NX pigs (false discovery rate < 0.05, Log_2_ fold change > 1.32 or Log_2_ fold change < −1.32, Figure 3F). HDCA and lithocholic acid (LCA) emerged as predominant BAs in the colonic contents of both pig types, with distinct distribution patterns, LCA species and muricholic acid species were significantly enriched in the colon of DLY pigs, whereas HCA species was significantly enriched in NX pigs (Figure S4B). In the DLY pigs, the LCA species, including LCA, iso‐lithocholic acid (iso‐LCA), and glycolithocholic acid (GLCA), showed significant enrichment. Conversely, the HCA species was more abundant in NX pigs (Figure S4C). Further analysis revealed a negative association between the levels of LCA and GLCA and backfat thickness, suggesting that higher concentrations of these BAs might reduce fat deposition (Figure S4D). To identify specific microbes influencing LCA production, we conducted a Spearman correlation analysis between BAs and bacterial species. The analysis demonstrated that LCA and its derivatives were significantly, positively correlated with the relative abundance of *B. pseudocatenulatum* in the colon (Figure S4E). Taken together, we proposed that *B. pseudocatenulatum* acts against fat deposition by regulating secondary BA biosynthesis, especially LCA production.

### *B. pseudocatenulatum* attenuates excessive fat deposition and enhances secondary BA biosynthesis in HFD‐fed mice

**Figure 4:**
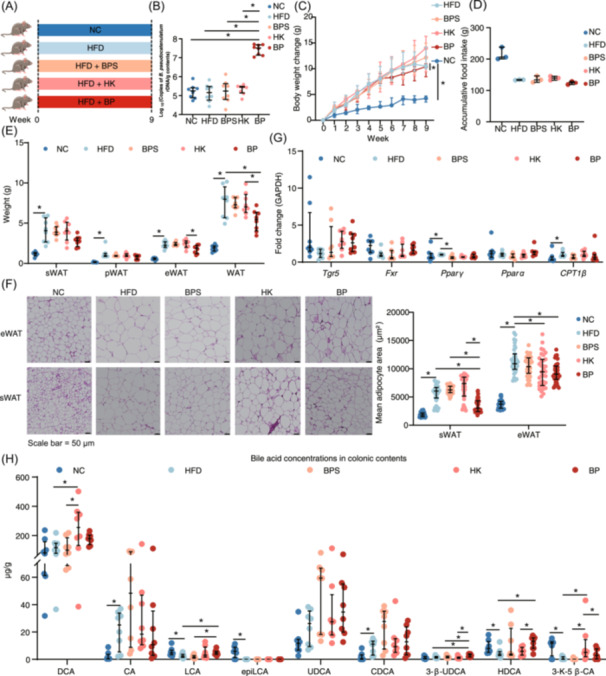
*Bifidobacterium pseudocatenulatum* attenuates excessive fat deposition and enhances secondary bile acid biosynthesis in high‐fat diet (HFD)‐fed mice. (A) Schematic of the *B. pseudocatenulatum* monocolonization experiment. C57BL/6 male mice were randomly divided into five groups: (1) negative control (NC) group: chow diet + vehicle; (2) HFD group: HFD + vehicle; (3) BPS group: HFD + *B. pseudocatenulatum* supernatant; (4) HK group: HFD + heat‐killed *B. pseudocatenulatum*; (5) BP group: HFD + live *B. pseudocatenulatum*. (B) The copies of *B. pseudocatenulatum* 16S rDNA in colonic contents. Median with interquartile range (Kruskal–Wallis test), *n* = 8 per group, *p < 0.05. (C) Body weight change. Median with interquartile range (friedman rank sum test), *n* = 13 per group, *p < 0.05. (D) Accumulative food intake. Median with range (Kruskal–Wallis test), *n* = 3 per group, *p < 0.05. (E) Adipose tissue weight. epididymal white adipose tissue (eWAT), perirenal white adipose tissue (pWAT), subcutaneous white adipose tissue (sWAT), white adipose tissue (WAT). Median with interquartile range (Kruskal–Wallis test), *n* = 8 per group, *p < 0.05. (F) Representative hematoxylin and eosin‐stained image of eWAT and sWAT, and quantification of the mean adipocytes area. *n* = 5 per group, *p < 0.05, Scale bar = 50 μm. (G) The mRNA expression of lipid metabolism in eWAT. Median with interquartile range (Kruskal–Wallis test), *n* = 8 per group, *p < 0.05. Takeda G protein‐coupled receptor 5 (*Tgr5*), Farnesoid X receptor (*Fxr*), Peroxisome proliferator‐activated receptor gamma (*Pparγ*), Peroxisome proliferator‐activated receptors α (*Pparα*), Carnitine palmitoyltransferase 1 beta (*Cpt1β*). (H) BA concentration in colonic contents. 3‐keto‐5*β*‐cholic acid (3‐K‐5*β*‐CA), 3*β*‐ursodeoxycholic acid (3‐*β*‐UDCA), cholic acid (CA), chenodeoxycholic acid (CDCA), deoxycholic acid (DCA), epilithocholic acid (epiLCA), hyodeoxycholic acid (HDCA), lithocholic acid (LCA), ursodeoxycholic acid (UDCA). Median with interquartile range (Kruskal–Wallis test), *n* = 8 per group, *p < 0.05. NC group: chow diet + vehicle; HFD group: HFD + vehicle; BPS group: HFD + *B. pseudocatenulatum* supernatant; HK group: HFD + heat‐killed *B. pseudocatenulatum*; BP group: HFD + live *B. pseudocatenulatum*. BA, bile acids.

To elucidate the role of *B. pseudocatenulatum* in fat deposition, we administered live *B. pseudocatenulatum*, heat‐killed *B. pseudocatenulatum*, *B. pseudocatenulatum* supernatant, and vehicle (BBL medium) to HFD‐fed mice for 9 weeks (Figure 4A). The colonization of the *B. pseudocatenulatum* had been confirmed by 16s rDNA quantification (Figure 4B). Only live *B. pseudocatenulatum* significantly decreased body weight change in HFD‐fed mice (Figure 4C,D and Figure S5A). Live *B. pseudocatenulatum* gavage also reduced the weight of total WAT, while decreased the mean adipocyte size of eWAT and sWAT (Figure 4E,F). Compared to HFD‐fed mice, live *B. pseudocatenulatum* treatment had no effect on *Fxr* and *Tgr5* expression (Figure 4G). In addition, live *B. pseudocatenulatum* also reduced liver weight, and alleviated hepatic steatosis, as evidenced by decreased serum alanine aminotransferase (ALT) levels (Figure S5B–E), indicating a protective effect against HFD‐induced liver injury. Furthermore, compared to HFD‐fed mice, live *B. pseudocatenulatum* and chow–diet treatment upregulated *Fxr* mRNA expression in liver (Figure S5F). To assess the impact on secondary BA metabolism, ultrahigh performance liquid chromatography‐mass spectra analysis was performed on the colonic contents. Compared to chow diet, HFD significantly increased colonic cholic acid (CA), and chenodeoxycholic acid (CDCA) concentrations, but decreased colonic LCA, epiLCA, HDCA, and 3‐keto‐5*β*‐cholic acid (3‐k‐5*β*‐CA) concentrations. Live *B. pseudocatenulatum* gavage significantly raised colonic LCA, 3*β*‐ursodeoxycholic acid (3‐β‐UDCA), and HDCA concentrations in HFD‐fed mice (Figure 4H).

Further experiments were conducted in antibiotic‐treated HFD‐fed mice to determine whether the anti‐fat deposition effect of *B. pseudocatenulatum* was independent of the gut microbiota. *B. pseudocatenulatum* consistently decreased body weight gain, fat mass, liver weight, and hepatic triglyceride concentrations in pseudo‐germ‐free HFD‐fed mice (Figure S6). These findings indicated that the anti‐fat deposition effect of *B. pseudocatenulatum* may be independent of the gut microbiota.

### Pharmacological inhibition of the BSH impairs the anti‐fat deposition effect of *B. pseudocatenulatum*

**Figure 5:**
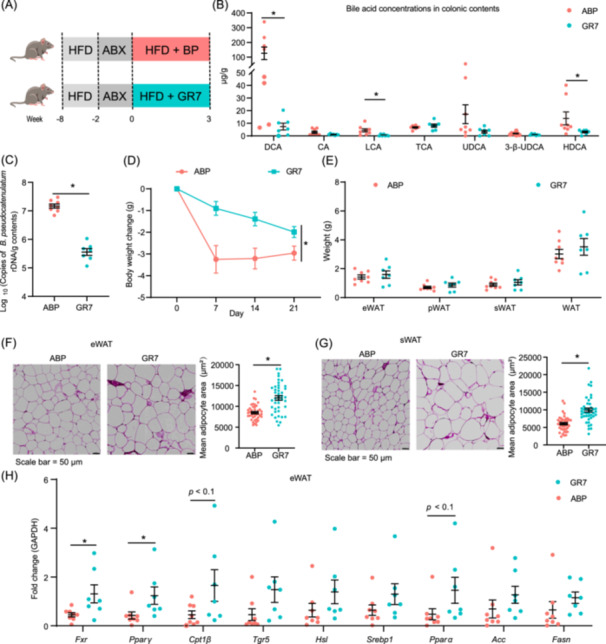
Pharmacological inhibition of the bile salt hydrolase impairs the anti‐fat deposition effect of *Bifidobacterium pseudocatenulatum* in antibiotic‐pretreated high‐fat diet (HFD)‐fed mice. (A) Schematic of pharmacological inhibition of bile salt hydrolase experiment. C57BL/6 male mice were pretreated with HFD for 8 weeks, and antibiotic cocktail for 2 weeks. Mice were gavage with *B. pseudocatenulatum* or *B. pseudocatenulatum* with GR7 for 3 weeks. ABP group: HFD + antibiotics mixture (ABX) + *B. pseudocatenulatum + *Vehicle (*n* = 8); GR7 group: HFD + ABX + *B. pseudocatenulatum *+ Gut‐restricted 7 (GR7, *n* = 7). (B) Colonic bile acids concentration. 3*β*‐ursodeoxycholic acid (3‐*β*‐UDCA), cholic acid (CA), deoxycholic acid (DCA), hyodeoxycholic acid (HDCA), lithocholic acid (LCA), taurocholic acid (TCA), ursodeoxycholic acid (UDCA). Data are shown as mean ± SEMs, ABP (*n* = 8), GR7 (*n* = 7), *p < 0.05 (Student's *t* test). (C) The copies of *B. pseudocatenulatum* 16S rDNA in colonic contents. Data are shown as mean ± SEMs, ABP (*n* = 8), GR7 (*n* = 7), *p < 0.05 (Student's *t* test). (D) Body weight change. Data are shown as mean ± SEMs, ABP (*n* = 8), GR7 (*n* = 7), *p < 0.05 (repeated measures analysis of variance). (E) Organ weight. epididymal white adipose tissue (eWAT), perirenal white adipose tissue (pWAT), subcutaneous white adipose tissue (sWAT), white adipose tissue (WAT). Data are shown as mean ± SEMs, ABP (*n* = 8), GR7 (*n* = 7), *p < 0.05 (Student's *t* test). (F) Representative hematoxylin and eosin (H&E) image of eWAT, and quantification of the mean adipocyte area. Data are shown as mean ± SEMs, ABP (*n* = 8), GR7 (*n* = 7), *p < 0.05 (Student's *t* test), Scale bar = 50 μm. (G) Representative H&E image of sWAT, and quantification of the mean adipocyte area. Data are shown as mean ± SEMs, ABP (*n* = 8), GR7 (*n* = 7), *p < 0.05 (Student's *t* test), Scale bar = 50 μm. (H) The mRNA expression of lipid metabolism in eWAT. Data are shown as mean ± SEMs, ABP (*n* = 8), GR7 (*n* = 7), *p < 0.05 (Student's *t* test). Farnesoid X receptor (*Fxr*), Peroxisome proliferator‐activated receptor gamma (*Pparγ*), Carnitine palmitoyltransferase 1 beta (*Cpt1β*), Takeda G protein‐coupled receptor 5 (*Tgr5*), Hormone‐sensitive lipase (*Hsl*), Sterol regulatory element‐binding protein 1 (*Srebp1*), Peroxisome proliferator‐activated receptors α (*Pparα*), Acetyl‐CoA carboxylase (*Acc*), Fatty acid synthase (*Fasn*). ABP group: HFD + ABX + live *B. pseudocatenulatum + *Vehicle; GR7 group: HFD + ABX + live *B. pseudocatenulatum* + GR7. SEM, standard error of the mean.

Using Proksee to predict genomic function, the *B. pseudocatenulatum* genome was found to contain a choloylglycine hydrolase (cgh) gene, which is involved in BA metabolism (Figure S7A). CGH, a bile salt hydrolase (BSH), catalyzes the conversion of conjugated BAs into free BAs. To determine whether the impact of *B. pseudocatenulatum* on fat deposition is mediated by BSH activity, we utilized the gut‐restricted BSH inhibitor GR7 in an HFD‐fed mouse model (Figure 5A). Co‐administration of *B. pseudocatenulatum* and GR7 revealed that GR7 significantly inhibited secondary BA biosynthesis (Figure 5B). GR7 treatment significantly decreased the colonization of *B. pseudocatenulatum* and reduced its impact on body weight (Figure 5C,D). Although GR7 treatment did not affect the weight of adipose tissue, it increased the mean adipocyte size of sWAT and eWAT (Figure 5E–G). Furthermore, GR7 exposure also increased serum glucose and triglyceride concentration (Figure S7B). Additionally, the treatment exacerbated liver injury, evidenced by elevated serum aspartate aminotransferase (AST) and ALT levels, and increased hepatic triglyceride content (Figure S7C,D). To further explore potential factors that may drive the alteration in lipid metabolism, we measured transcript levels of *Fxr*, *Pparγ*, and *Tgr5*. Administration of GR7 significantly downregulated liver *Fxr*, and upregulated *Fxr*, and *Pparγ* expression in eWAT (Figure 5H and Figure S7E). Taken together, these findings underscore the impact of BSH activity on fat deposition.

### LCA protects against fat deposition in HFD‐fed rat and NX pig models

**Figure 6:**
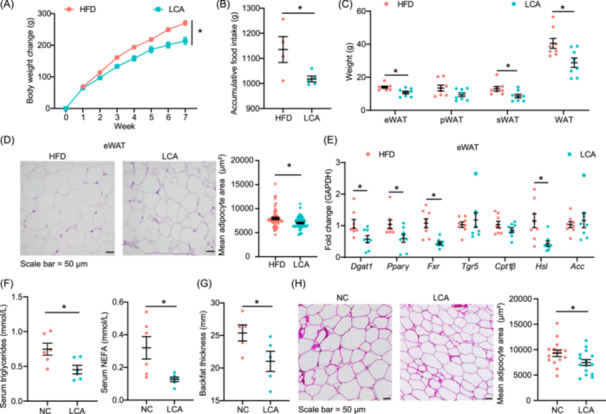
Dietary supplementation with lithocholic acid (LCA) against fat deposition in high‐fat diet (HFD) fed rats and Ningxiang pigs. (A) Body weight change of rats. Data are shown as mean ± SEMs, *n* = 8 per group, *p < 0.05 (repeated measures analysis of variance). HFD group: rats fed an HFD; LCA group: rats fed an HFD supplemented with 0.3% LCA. (B) Accumulative food intake of rats. Data are shown as mean ± SEMs, *n* = 4 per group, *p < 0.05 (Student's *t* test). HFD group: rats fed an HFD; LCA group: rats fed an HFD supplemented with 0.3% LCA. (C) Adipose tissue weight of rats. epididymal white adipose tissue (eWAT), perirenal white adipose tissue (pWAT), subcutaneous white adipose tissue (sWAT), white adipose tissue (WAT). Data are shown as mean ± SEMs, *n* = 8 per group, *p < 0.05 (Student's *t* test). HFD group: rats fed an HFD; LCA group: rats fed an HFD supplemented with 0.3% LCA. (D) Representative hematoxylin and eosin (H&E)‐stained image of eWAT, and quantification of the mean adipocytes area in rats. Data are shown as mean ± SEMs, *n* = 8 per group, *p < 0.05 (Student's *t* test), Scale bar = 50 μm. HFD group: rats fed an HFD; LCA group: rats fed an HFD supplemented with 0.3% LCA. (E) The mRNA expression of lipid metabolism in eWAT of rats. Data are shown as mean ± SEMs, *n* = 8 per group. *p < 0.05 (Student's *t* test). HFD group: rats fed an HFD; LCA group: rats fed an HFD supplemented with 0.3% LCA. Diacylglycerol O‐acyltransferase 1 (*Dgat1*), Peroxisome proliferator‐activated receptor gamma (*Pparγ*), Farnesoid X receptor (*Fxr*), Takeda G protein‐coupled receptor 5 (*Tgr5*), Carnitine palmitoyltransferase 1 beta (*Cpt1β*), Hormone‐sensitive lipase (*Hsl*), Acetyl‐CoA carboxylase (*Acc*). (F) Serum triglycerides, and nonesterified fatty acid concentrations of rats. Data are shown as mean ± SEMs, *n* = 6 per group. *p < 0.05 (Student's *t* test). Negative control (NC) group: pigs fed a basal diet; LCA group: pigs fed a basal diet supplemented with 792 mg/kg LCA. (G) Backfat thickness of NX pigs. Data are shown as mean ± SEMs, *n* = 5 per group. *p < 0.05 (Student's *t* test). NC group: pigs fed a basal diet; LCA group: pigs fed a basal diet supplemented with 792 mg/kg LCA. (H) Representative H&E‐stained image of subcutaneous adipose tissue and quantification of the mean adipocytes area in NX pigs. Data are shown as mean ± SEMs, *n* = 3 per group. *p < 0.05 (Student's *t* test), Scale bar = 50 μm. NC group: pigs fed a basal diet; LCA group: pigs fed a basal diet supplemented with 792 mg/kg LCA. SEM, standard error of the mean.

The monocolonization experiment with *B. pseudocatenulatum* and pharmacological inhibition of BSH consistently influenced colonic LCA concentrations in HFD‐fed mice. To investigate the effect of LCA on fat deposition, LCA (0.3% in diet) was supplemented into the HFD. Compared to the HFD group, the LCA group significantly decreased body weight gain and accumulative food intake (Figure 6A,B). LCA supplementation also led to a reduction in serum triglycerides concentrations and an increase in serum LDLC concentrations, correlating enhanced activity of serum lipoprotein lipase (LPL), an enzyme involved in triglyceride hydrolysis (Figure S8A,B). On a tissue level, LCA significantly decreased the weights of eWAT, sWAT, and WAT, alongside a reduction in the mean adipocyte area of eWAT (Figure 6C,D). Transcript analysis revealed that LCA supplementation downregulated the mRNA expression of diacylglycerol O‐acyltransferase 1, *Fxr*, Hormone‐sensitive lipase (*Hsl*), and *Pparγ* in eWAT (Figure 6E). In addition, LCA significantly reduced liver weight and hepatic steatosis scores (Figure S8C,D). Transcript analysis showed LCA upregulated the expression of *Fxr* and downregulated the expression of *Fasn* and *Acc* in liver (Figure S8E).

Furthermore, we conducted a dietary intervention experiment in NX pigs. Compared to the negative control (NC) group, LCA did not affect body weight change but significantly decreased backfat thickness, as well as serum triglyceride and NEFA concentrations in NX pigs (Figure 6F,G and Figure S8F). LCA supplementation also notably decreased hepatic NEFA concentrations, which aligned with downregulated expressions of *Pparγ* and *Srebp1* and upregulated carnitine palmitoyl transferase 1 beta in the liver of NX pigs (Figure S8G,H). In addition, LCA supplementation significantly reduced the mean adipocyte area of SAT (Figure 6H).

## DISCUSSION

In the present study, DLY and NX pigs were used as models to investigate the effect of gut microbiota on fat deposition. Here, we demonstrated that the effect of colonic microbiota on fat deposition varies between obese and lean pigs, with obese pig's microbiota specifically enhancing fat accumulation. Furthermore, integrated metagenomic and metabolomic analyses indicated that *B. pseudocatenulatum* may improve fat deposition by regulating secondary BA metabolism. *B. pseudocatenulatum* was found to effectively attenuate fat deposition and hepatic steatosis in HFD‐fed mice. Mechanistically, *B. pseudocatenulatum* was shown to enhance secondary BA biosynthesis, while pharmacological inhibition of BSH impaired the anti‐fat deposition effects of *B. pseudocatenulatum*. Additionally, LCA exhibited similar anti‐fat deposition effects in HFD‐fed rats and NX pigs.

The use of germ‐free animals and fecal microbiota transplantation has highlighted the critical role of gut microbiota in fat deposition. Previous studies in humans and mice have demonstrated significant differences in gut microbiota composition between obese and healthy individuals [17, 18], and the gut microbiota in obese individuals has been shown to enhance energy harvest from the diet [4]. Our study also revealed a significant difference in gut microbiota composition between DLY and NX pigs, with the lipid metabolism pathway being enriched in the colonic microbiota of NX pigs. Fecal microbiota transplantation from obese twins has been shown to increase fat deposition in mice [4]. Transplantation of fecal microbiota from Jinhua pigs and Landrace pigs into broad‐spectrum antibiotic‐treated mice demonstrated that mice receiving Jinhua pig's fecal microbiota exhibited increased fat deposition in the liver and abdominal fat [19]. In our experiments, we transplanted colonic microbiota from DLY and NX pigs into chow– diet‐fed and HFD‐fed mice. The results indicated that mice receiving NX pig's colonic microbiota showed increased fat deposition in the liver and WAT when fed an HFD. Additionally, compared to mice receiving DLY pig's colonic microbiota, those receiving NX pig's colonic microbiota had decreased serum total BA concentrations. Although genetic background may affect the outcomes of microbiota transplantation experiments, the results of cross‐species microbiota transplantation are still widely recognized and accepted [20, 21]. These findings suggested that the colonic microbiome derived from NX pigs has a stronger capacity to induce host fat deposition compared to that derived from DLY pigs.

Some gut microbiota, such as *Akkermansia muciniphila* and *Bifidobacterium* spp., have been shown to regulate host fat storage. Zhao et al. [7] demonstrated through fecal shotgun metagenomics that *B. pseudocatenulatum* improves glucose homeostasis. Additionally, our shotgun metagenomics analysis revealed a negative association between colonic *B. pseudocatenulatum* and backfat thickness. Interestingly, several studies have reported a high relative abundance of *B. pseudocatenulatum* that is negatively associated with obesity [22, 23]. In the present study, gavaging live *B. pseudocatenulatum* also decreased fat deposition and hepatic steatosis in HFD‐fed mice. However, gavaging the supernatant and heat‐killed *B. pseudocatenulatum* failed to reduce fat deposition and hepatic steatosis. This suggests that the regulatory effect of *B. pseudocatenulatum* on fat deposition is likely due to the crosstalk between metabolic processes and live *B. pseudocatenulatum*. The failure of the supernatant and heat‐killed bacteria to produce similar effects could be attributed to the instability or insufficient concentration of critical metabolites, or the absence of direct interactions between live *B. pseudocatenulatum* and the host, which are essential for modulating lipid metabolism. Secondary BA metabolism differs significantly between in vivo and in vitro, notably, BAs play a crucial role in lipid metabolism [12, 24]. In addition, we found that LCA species were significantly enriched in DLY pigs and positively associated with *B. pseudocatenulatum*. Gavage with *B. pseudocatenulatum* increased fecal LCA concentration in a collagen‐induced arthritis mouse model [25]. Consistent with this study, gavage administration with live *B. pseudocatenulatum* also increased colonic LCA concentration in HFD‐fed mice. These results indicate that *B. pseudocatenulatum* may mitigate fat deposition by enhancing secondary BA biosynthesis. BSH medicates secondary BA biosynthesis, and Targoński et al [26] purified BSH from *B. pseudocatenulatum*. However, the role of BSH in lipid metabolism remains unknown. Delvin et al. [27] developed a gut‐restricted inhibitor of BSH, therefore, we used it in the present study. Pharmacological inhibition of BSH with GR7 increased serum triglycerides, mean adipocyte area, and liver steatosis score, while decreasing the colonization of *B. pseudocatenulatum* in the colon of HFD‐fed mice. These results are consistent with findings that colonizing mice with another bacterium exogenously expressing bile‐modifying genes from *Turicibacter* strains decreases serum cholesterol, triglycerides, and adipose tissue mass [28]. Expressed BSH in *Escherichia coli* also significantly decreased weight gain and improved lipid metabolism in mice [29]. However, some studies have indicated that inhibiting BSH may alleviate diseases, such as nonalcoholic fatty liver disease or obesity [30, 31, 32]. The differences observed here warrant further investigation in future studies. BAs are saturated, hydroxylated C‐24 cyclopentane phenanthrene sterols synthesized from cholesterol in hepatocytes. The ratio of non‐12‐OH BAs to 12‐OH BAs is negatively associated with body mass index in HFD‐fed mice, and LCA belongs to non‐12‐OH BAs [33]. LCA is elevated following sleeve gastrectomy to improve diabetes by the production of cholic acid‐7‐sulfate [34]. In the present study, dietary supplementation with LCA decreased fat deposition and hepatic steatosis in rats and NX pigs. However, one study has indicated that LCA can worsen glucose tolerance and impair systemic metabolism in chow–diet‐fed mice [35]. We speculate this may be related to the physiological state of the mice and the concentration of LCA. Previous research has established that LCA acts as an agonist for both *Fxr* and *Tgr5*, and the upregulation of these receptors has been shown to mitigate fat deposition. Our study corroborates these findings, demonstrating that LCA alleviates hepatic steatosis while upregulating hepatic *Fxr* and *Tgr5* expression, suggesting that LCA may reduce fat accumulation through these pathways. However, in adipose tissue, LCA did not affect *Fxr* and *Tgr5* expression but instead downregulated *Pparγ*. This indicates that *Fxr*, *Tgr5*, and *Pparγ* could be potential targets of LCA, though this hypothesis requires further validation in gene knockout models. Although our study has demonstrated that LCA can reduce fat deposition, current mechanistic studies remain limited. Future research should focus on elucidating the mechanisms underlying this effect.

There are several limitations in the present study. First, NX and DLY pigs have different genetic background, and the effect of genetic background on gut microbiota is not well understood. Second, the sex‐based effect in gut microbiome and BAs profilings were ignored. Third, overexpression of the BSH of *B. pseudocatenulatum* and germ‐free mice studies need to be performed in future study. In addition, during each cycle of the BA's enterohepatic circulation, about 95% of BAs are recovered in the gut and the 5% that enter the colon are converted into secondary BAs. Therefore, in subsequent experiments, a more comprehensive consideration of BA changes in the ileum may help us better understand this issue.

## CONCLUSION

Taken together, our study demonstrates that the effects of colonic microbiota on fat deposition varies between obese and lean pigs. Through shotgun metagenomics and metabolomics, we identify that *B. pseudocatenulatum* and LCA species are significantly enriched in the colons of DLY pigs. Moreover, gavage with live *B. pseudocatenulatum* reduces fat deposition in the liver and adipose tissue, while increases colonic LCA concentration. Pharmacological inhibition of the BSH of *B. pseudocatenulatum* leads to increased fat deposition in HFD‐fed mice. In addition, dietary supplementation with LCA also decreases fat deposition in HFD‐fed rats and NX pigs. These results proved *B. pseudocatenulatum* reduces fat deposition by enhancing secondary BA biosynthesis.

## METHODS

### Experimental animals and treatments

#### Animal experiment 1: DLY and NX pigs sample collection

Forty DLY pigs (Tangrenshen Group Co., Ltd.) and forty NX pigs (Chuweixiang Co., Ltd.) with approximately 100 kg body weight were used in this study. The diets for NX and DLY pigs are specifically formulated to address their distinct nutritional requirements (Tables S1 and S2), and all pigs had free access to feed and water. Tissue samples were rapidly collected after sacrifice. Backfat thickness was measured using a vernier caliper at the junction area of the 6th and 7th thoracic vertebrae.

#### Animal experiment 2: Colonic microbiota transplant experiment in chow–diet‐fed mice

Colonic microbiota suspensions were prepared according to previously described methods [36, 37]. Briefly, fresh colonic contents were collected from healthy DLY and NX pigs, homogenized in sterile saline, filtered through a sterile stainless‐steel strainer, and centrifuged to remove insoluble matter. The OD_600_ of the suspension was adjusted to 1.0 with sterile saline solution, and 10% sterile glycerol (final concentration) was added before storage at ‐80°C.

The experimental design is shown in Figure 2A. Mice (C57BL/6 J, SLAC, Shanghai, China) aged 7–8 weeks were administered a mixture of antibiotics (vancomycin 0.5 g/L, neomycin 1 g/L, metronidazole 1 g/L, ampicillin 1 g/L, all from Sigma) in their drinking water for 2 weeks, as previously described [38]. The mice were then randomly assigned into two groups and treated with different colonic bacterial suspensions. (1) DLY‐CD group: received 200 µL colonic bacterial suspension from DLY pigs (*n* = 8); (2) NX‐CD group: received 200 µL colonic bacterial suspension from NX pigs (*n* = 8) [7, 39]. All mice were fed standard chow diet (10% energy from fat; 3.25 kcal/g; SLAC). After 8 weeks, eight mice from each group were euthanized, and liver, adipose tissue, and serum samples were collected for further analysis.

#### Animal experiment 3: Colonic microbiota transplant experiment in HFD‐fed mice

The experimental design is shown in Figure 2A. Mice (C57BL/6 J, SLAC) aged 7–8 weeks were administered a mixture of antibiotics (vancomycin 0.5 g/L, neomycin 1 g/L, metronidazole 1 g/L, ampicillin 1 g/L, all from Sigma) in their drinking water for 2 weeks, as previously described [38]. The mice were then randomly assigned to two groups and treated with different colonic bacterial suspensions: (1) DLY‐HFD group: received colonic bacterial suspension from DLY pigs (*n* = 8); (2) NX‐HFD group: received colonic bacterial suspension from NX pigs (*n* = 8). All mice were fed an HFD, 60% from fat, D12492, Research Diet). After 8 weeks, eight mice from each group were euthanized, liver, adipose tissue, and serum samples were collected for further analysis.

#### Animal experiment 4: *B. pseudocatenulatum* monocolonization experiment

The experimental design is shown in Figure 4A. *B. pseudocatenulatum* (CGMCC 1.2277) was obtained from the China General Microbiological Culture Collection Center. *B. pseudocatenulatum* was cultured in sterile BBL medium (MM1921, Coolaber) at 37°C for 48 h in the anaerobic chamber (85% N_2_:10% H_2_:5% CO_2_). Healthy male mice, aged 7‐8 weeks, were randomly divided into five groups: (1) NC group: mice fed a chow diet (*n* = 13); (2) HFD group: mice fed an HFD (*n* = 13); (3) BPS group: mice fed an HFD, and gavaged with 200 µL *B. pseudocatenulatum* supernatant (*n* = 13); (4) HK group: mice fed an HFD group, and orally gavaged with 200 µL heat‐killed *B. pseudocatenulatum* (1 × 10^8 ^colony‐forming unit (CFU)/200 µL BBL medium) (*n* = 13); (5) BP group: mice fed an HFD, and orally gavaged with 200 µL live *B. pseudocatenulatum* (1×10^8^ CFU/200 µL BBL medium) (*n* = 13). After 9 weeks, eight mice from each group were euthanized, liver, adipose tissue, colonic, and serum samples were collected for further analysis.

#### Animal experiment 5: *B. pseudocatenulatum* monocolonization in pseudo germ free mice

The experimental design is shown in Figure S6A. Healthy male mice (C57BL/6J, SLAC), aged 7–8 weeks were administered a mixture of antibiotics (vancomycin 0.5 g/L, neomycin 1 g/L, metronidazole 1 g/L, ampicillin 1 g/L, all from Sigma) in their drinking water for 2 weeks, as previously described [38]. The mice were then randomly divided into two groups: (1) ABX group: mice fed an HFD diet (*n* = 6) and orally gavaged with 500 µL BBL medium; (2) ABP group: mice were fed HFD and orally gavaged with 500 µL live *B. pseudocatenulatum* (5 × 10^8 ^CFU/500 µL BBL medium) (*n* = 6). After 5 weeks, six mice from each group were euthanized, liver, adipose tissue, colonic, and serum samples were collected for further analysis.

#### Animal experimental 6: Pharmacological inhibition the BSH of *B. pseudocatenulatum*

The experimental design is shown in Figure 5A. Eight‐week‐old male C57BL/6J mice were fed an HFD for 8 weeks and then gavaged with 100 μL of an antibiotic cocktail containing vancomycin (0.5 g/L, Sigma), neomycin (0.5 g/L, Sigma), metronidazole (1 g/L, Sigma), and ampicillin (1 g/L, Sigma) for 14 days [40]. Microbiota depletion was verified by aerobic and anaerobic culture of fecal pellets. The mice were then randomly assigned into two groups: (1) ABP group: mice fed an HFD, and orally gavaged with 500 µL live *B. pseudocatenulatum* (5 × 10^8^ CFU/500 µL BBL medium) (*n* = 8) [41]; (2) GR7 group: mice fed an HFD, orally gavaged with 500 µL live *B. pseudocatenulatum* (5 × 10^8 ^CFU/500 µL BBL medium) and 10 mg/kg gut‐restricted 7 (GR‐7, HY‐135747, MedChemExpress, Shanghai, China) (*n* = 7) [27]. After 3 weeks, fifteen mice were euthanized, liver, adipose tissue, colonic, and serum samples were collected for further analysis.

#### Animal experiment 7: LCA treatment experiment in rat

Eight‐week‐old healthy male rat (SLAC) were randomly assigned into two groups based on body weight: (1) HFD group: rats fed an HFD (*n* = 8); (2) LCA group: rats fed an HFD supplemented with 0.3% LCA (L106779, aladdin) (*n* = 8). The dose of LCA refers to previous study [42]. Rats were housed under a 12‐h light‐dark cycle. The experiment lasted for 7 weeks. Liver, adipose tissue, colonic, and serum samples were collected for further study.

#### Animal experiment 8: LCA treatment experiment in NX pig model

A total of 12 pigs were randomly assigned into two groups based on body weight: (1) NC group: pigs fed a basal diet; (2) LCA group: pigs fed a basal diet supplemented with 792 mg/kg LCA (Yinfeng biological). The basal diet formula is shown in Table S3 and meets the nutrient requirements of NX pigs. All pigs had free access to water and feed. The dose of LCA based on the colonic LCA concentration of DLY pigs. Five pigs from each group were euthanized after 10 weeks, liver, adipose tissue, colonic, and serum samples were collected for further study.

### DNA extraction and 16S rRNA gene sequencing

Bacterial DNA from colonic contents was extracted using Mobio PowerSoil R DNA extraction kit (Zymo Research) according to manufacturer instructions. Extracted DNA was assessed using 2% agarose gel electrophoresis and Nanodrop‐1000 (Thermo Scientific). The barcode primers were 341 F (5′‐CCTACGGGNGGCWGCAG‐3′), and 805 R (5′‐GACTACHVGGGTATCTAATCC‐3′). These primers were used to amplify the V3–V4 hypervariable region of the 16S rRNA. The purified PCR amplification products were used to construct sequencing library. All amplicons were sequenced by pair‐end method on Novaseq platform (Illumina). The sequence data can be found in SRA database (PRJNA816269). A total of 6,113,705 raw tags, with 76,421 ± 57 raw tags per sample, were obtained by FLASH (version 1.2.7) [43]. A total of 5,763,836 clean tags, with 72,048 clean tags per sample were obtained by Trimmomatic (v. 0.33) [44]. Finally, effective tags were obtained using UCHIME (v. 4.2) to exclude chimeras [45]. Effective tags were processed according to quantitative insights into microbial ecology (QIIME, v. 1.9.1) pipeline, and cluster into operational taxonomic units (OTUs) at a 97% similarity with open reference algorithm. Taxonomies were obtained using ribosomal database project classifier aligned to the Greengenes database (May 2013 release) [46]. Diversity analysis was performed based on OTU table and feature sequences. Alpha diversity was calculated by observed OTUs, Shannon, Simpson, and Pielou indices. Beta diversity was calculated based on Bray–curtisdistance. The sequence data can be found in National Center for Biotechnology Information (NCBI) (GSA: PRJNA816269).

### Metagenomic sequencing analysis

DNA sequencing libraries were prepared with the TruSeq Nano DNA LT library preparation kit—Set A (FC‐121‐4001, Illumina) and nuclease‐free water (AM9932, Ambion). High‐throughput sequencing was performed on the NovaSeq. 6000 platform (PE150, Illumina) after validating the sequencing libraries (LC‐Bio).

Raw sequencing data were processed to generate clean data for in‐depth analyses. Adapter trimming was conducted using Trim Galore (v. 0.6.5) with the following parameters: removal of reads shorter than 75 bp, trimming of bases with a quality score lower than 20, and removal of adapter sequences [47]. Host genomic sequences were removed using BMTagger, with reference to the *Sus scrofa* 11.1 genome (NCBI accession number GCA_000003025.6) [48]. Quality control checks on the processed data were performed using FastQC (v. 0.11.9). Taxonomic classification was achieved using Kaiju aligned with the NCBI RefSeq database. Open reading frames were predicted using Prodigal (v. 2.6.3) [49]. Functional annotation was carried out using Diamond (v. 2.0.15) with a cutoff E‐value of ≤10^−10^, aligned with the KEGG database (https://www.kegg.jp/). The sequence data can be found in China National Center for Bioinformation (accession number PRJCA022867).

### Colonic metabolomics

Colonic metabolomics were analyzed using UPLC‐MS/MS (ACQUITY UPLC‐Xevo TQ‐S, Waters Corp.). All metabolite standards were obtained from Sigma‐Aldrich, Steraloids Inc., and TRC Chemicals. The raw data files generated by UPLC‐MS/MS were processed using Mass Lynx software (v. 4.1, Waters). The self‐developed platform iMAP (Metabo‐Profile) was used for statistical analyses, including principal component analysis, univariate analysis, and pathway analysis.

### Quantification of *B. pseudocatenulatum*

This experiment was performed according to the previous study [50, 51]. Total DNA from colonic contents was extracted using a DNA Kit (Vazyme). *B. pseudocatenulatum* was quantified with species‐specific primers (BiCAT‐F: 5′‐CGGATGCTCCGACTCCT‐3′, BiCAT‐R: 5′‐CGAAGGCTTGCTCCCGAT‐3′) [52]. A real‐time fluorescence quantification kit (SYBR Green, Accurate Biotechnology Co., Ltd.) was used to detect the copies.

### Determination of serum biochemical indexes

Serum ALT, AST, alkaline phosphatase, total BA, glucose, triglyceride, cholesterol, LDLC, HDLC, and NEFA concentrations were measured using an automatic biochemistry analyzer and assay kits (Roche). Hepatic triglyceride levels were measured using a triglyceride assay kit (NJJCBIO) according to manufacturer instructions.

### Histological analysis of liver and WAT

Hematoxylin and eosin (H&E) staining was performed as previously described [53]. Tissue samples were collected after sacrifice and fixed in 4% paraformaldehyde solution. Fixed samples were dehydrated with ethanol, embedded with paraffin, stained with H&E, and sealed with neutral balsam. Hepatic steatosis assessment was carried out using digital microscope (BA210Digital, Motic) as previously described [54]. Adipocytes size was measured by Motic Images Advanced software. The hepatic steatosis score was evaluated according to previously study [55]. Adipocyte cell was outlined by enhanced contrast function, and adipocyte area size was measured by ImageJ 6.0.

### Determination of LPL activity

Tissue samples were homogenized, and protein concentration was determined using a BCA protein assay kit (Beyotime Biotechnology). LPL activity was measured using assay kits (CUSABIO) as previously described [56], with results normalized to protein concentration.

### Functional annotation of bacterial genomes

The genomic sequences of *B. pseudocatenulatum* (GCA_001025215.1) were downloaded from NCBI. Functional annotation of the bacterial genome was conducted using Proksee. Genomic sequences were uploaded for automated annotation using integrated tools such as Prokka.

### Secondary BAs determination

Colonic BA concentrations were measured according to previous studies [57, 58]. For sample pretreatment, 20 mg colonic contents were mixed with 1 mL methanol‐water solution (v/v = 5:5) containing 2% internal standards (D4‐CA, D4‐LCA,60 µg/mL each). The mixture underwent ultrasonic extraction at 60 Hz for 10 min, followed by centrifugation at 13,000 g at 4°C for 10 min. The supernatant was collected, and the extraction procedure was repeated three times. Then 700 µL of the pooled supernatant was processed using solid‐phase extraction, followed by filtration through a 0.22 µm filter membrane. The final supernatant was transferred to a 1 mL vial for analysis by UPLC‐MS. The secondary BAs detected in this study included CA, taurocholic acid, deoxycholic acid (DCA), glycocholic acid, UDCA, taurodeoxycholic acid, glycodeoxycholic acid, 3*β*‐UDCA, HDCA, iso‐deoxycholic acid, LCA, epi‐lithocholic acid, iso‐LCA, 3α‐hydroxy‐5*α*‐cholanic acid, 7‐ketolithocholic acid, 12‐ketolithocholic acid, 5α‐cholanic acid‐3*α*‐ol, glycochenodeoxycholic acid, glycoursodeoxycholic acid, taurochenodeoxycholic acid, tauroursodeoxycholic acid, CDCA, glycohyodeoxycholic acid, taurohyodeoxycholic acid, allocholic acid, and taurolithocholic acid.

### Real‐time quantitative PCR

The experiment was conducted as previously described [59]. Briefly, tissue samples were ground into powder, and total RNA was extracted using the TRIZOL (15596‐026, Invitrogen) method. Then, the concentration and quality of the total RNA were measured using a Nanodrop ND‐1000 (Thermo Scientific). The total RNA was then reverse‐transcribed into cDNA by reverse transcription kit. The primers are showed in Table S4. A real‐time fluorescence quantification kit (SYBR Green, Accurate Biotechnology Co., Ltd.) was used to detect the mRNA expression of target genes, with glyceraldehyde 3‐phosphate dehydrogenase (*Gapdh*) serving as the housekeeping gene to normalize gene expression.

### Statistical analysis

Data were organized using Excel 2016. Two‐way repeated‐measures analysis of variance (ANOVA) was used to compare trends across two curves over multiple time points. The Friedman test was used for five‐group comparisons with repeated measures over time. The two groups were compared using a two‐tailed unpaired Student's *t* test as appropriate. For multigroup comparisons, one‐way ANOVA was used for normally distributed data, while the Kruskal–Wallis test was used for nonnormally distributed data. Statistical analysis was conducted with IBM SPSS Statistics 22.0 software (IBM Corp. Released 2013. IBM SPSS Statistics for Windows, version 22.0.: IBM Corp.), with significance set at *p* < 0.05. Data visualization was carried out using R (v. 4.1.2) and Graphpad prism 8.0 software. Additional data analyses, including PCoA, mantel tests, LDA LEfSe, and spearman correlation analysis were conducted in RStudio (v 2024.04.2). Random forest analysis was performed using the “randomForest” package in R (v. 4.1.2).

## AUTHOR CONTRIBUTIONS

**Andong Zha**: Conceptualization; methodology; software; data curation; formal analysis; investigation; writing—original draft; writing—review and editing; visualization; validation. **Ming Qi**: Conceptualization; methodology; software. **Yuankun Deng**: Conceptualization; methodology; software; data curation. **Hao Li**: Conceptualization; methodology; investigation. **Nan Wang**: Conceptualization; methodology; investigation; software. **Chengming Wang**: Conceptualization; methodology; software. **Simeng Liao**: Methodology; software. **Dan Wan**: Conceptualization; methodology; investigation. **Xia Xiong**: Conceptualization; methodology. **Peng Liao**: Conceptualization; methodology; software. **Jing Wang**: Funding acquisition; project administration; resources; supervision. **Yulong Yin**: Funding acquisition; project administration; supervision; writing—review and editing; resources. **Bi'e Tan**: Funding acquisition; project administration; supervision; resources; writing—review and editing.

## CONFLICT OF INTEREST STATEMENT

The authors declare no conflicts of interest.

## ETHICS STATEMENT

The experimental methods and procedures were approved by the Institutional Animal Care and Use Committee of Hunan Agricultural University (No. 202003). The experiment complied with the ARRIVE guidelines.

## Supporting information


**Figure S1.** Comparison of lipid metabolism pathways in the colonic microbiota.
**Figure S2.** The impact of the colonic microbiome on hepatic fat deposition varies among different breed.
**Figure S3.** Identification of differential bacterial biomarkers between Duroc × Landrace × Yorkshire and Ningxiang pigs by linear discriminant analysis effect size.
**Figure S4.** Colonic microbiome mediates variations in secondary bile acid metabolism between lean and obese pigs.
**Figure S5.** *Bifidobacterium pseudocatenulatum* attenuates hepatic steatosis in high‐fat diet‐fed mice.
**Figure S6.** *Bifidobacterium pseudocatenulatum* attenuates fat deposition in antibiotic‐pretreated high‐fat diet fed mice.
**Figure S7.** GR7 impairs *Bifidobacterium pseudocatenulatum* attenuates hepatic steatosis in high‐fat diet ‐fed mice.
**Figure S8.** Lithocholic acid attenuates hepatic steatosis in high‐fat diet‐fed rats and Ningxiang pigs.

**Table S1**. The ingredients and nutrition levels of Duroc × Landrace × Yorkshire pig's feed (%, as‐fed basis).
**Table S2**. The ingredients and nutrition levels of Ningxiang pig's feed (%, as‐fed basis).
**Table S3**. The ingredients and nutrition levels of the basal diet (%, as‐fed basis).
**Table S4**. Primers used in this study.

## Data Availability

The raw sequence data of metagenomic reported in this paper have been deposited in the Genome Sequence Archive (Genomics, Proteomics & Bioinformatics 2021) in National Genomics Data Center (Nucleic Acids Res 2022), China National Center for Bioinformation/Beijing Institute of Genomics, Chinese Academy of Sciences (GSA: CRA014539) that are publicly accessible at http://ngdc.cncb.ac.cn/gsa. The raw sequence data of 16S rRNA sequencing reported in this paper have been deposited in the NCBI (GSA: PRJNA816269, http://www.ncbi.nlm.nih.gov/bioproject/PRJNA816269/). The data and scripts are available on GitHub: http://github.com/ZAD-Vitamin/ZhaiMeta. Supplementary materials (figures, tables, graphical abstract, slides, videos, Chinese translated version, and update materials) may be found in the online DOI or iMeta Science http://www.imeta.science/.

## References

[1] Després, Jean‐Pierre , and Isabelle Lemieux . 2006. “Abdominal Obesity and Metabolic Syndrome.” Nature 444: 881–887. 10.1038/nature05488 17167477

[2] Chen, Congying , Shaoming Fang , Hong Wei , Maozhang He , Hao Fu , Xinwei Xiong , Yunyan Zhou , et al. 2021. “Prevotella Copri Increases Fat Accumulation in Pigs Fed With Formula Diets.” Microbiome 9: 175. 10.1186/s40168-021-01110-0 34419147 PMC8380364

[3] Turnbaugh, Peter J. , Fredrik Bäckhed , Lucinda Fulton , and Jeffrey I. Gordon . 2008. “Diet‐Induced Obesity Is Linked to Marked But Reversible Alterations in the Mouse Distal Gut Microbiome.” Cell Host & Microbe 3: 213–223. 10.1016/j.chom.2008.02.015 18407065 PMC3687783

[4] Turnbaugh, Peter J. , Ruth E. Ley , Michael A. Mahowald , Vincent Magrini , Elaine R. Mardis , and Jeffrey I. Gordon . 2006. “An Obesity‐Associated Gut Microbiome With Increased Capacity for Energy Harvest.” Nature 444: 1027–1031. 10.1038/nature05414 17183312

[5] Fan, Yong , and Oluf Pedersen . 2021. “Gut Microbiota in Human Metabolic Health and Disease.” Nature Reviews Microbiology 19: 55–71. 10.1038/s41579-020-0433-9 32887946

[6] Sroka, Oleksiak A. , Agata Młodzińska , Małgorzata Bulanda , Dominika Salamon , Piotr Major , Maciej Stanek , and Tomasz Gosiewski . 2020. “Metagenomic Analysis of Duodenal Microbiota Reveals a Potential Biomarker of Dysbiosis in the Course of Obesity and Type 2 Diabetes: A Pilot Study.” Journal of Clinical Medicine 9(2): 369. 10.3390/jcm9020369 32013181 PMC7074165

[7] Zhao, Liping , Feng Zhang , Xiaoying Ding , Guojun Wu , YanY Lam , Xuejiao Wang , Huaqing Fu , et al. 2018. “Gut Bacteria Selectively Promoted by Dietary Fibers Alleviate Type 2 Diabetes.” Science 359: 1151–1156. 10.1126/science.aao5774 29590046

[8] Mariño, Eliana , James L. Richards , Keiran H. McLeod , Dragana Stanley , Yu Anne Yap , Jacinta Knight , Craig McKenzie , et al. 2017. “Gut Microbial Metabolites Limit the Frequency of Autoimmune T Cells and Protect Against Type 1 Diabetes.” Nature Immunology 18: 552–562. 10.1038/ni.3713 28346408

[9] Fernández, Veledo S. , Victòria Ceperuelo‐Mallafré , and Joan Vendrell . 2021. “Rethinking Succinate: An Unexpected Hormone‐Like Metabolite in Energy Homeostasis.” Trends in Endocrinology & Metabolism 32: 680–692. 10.1016/j.tem.2021.06.003 34301438

[10] Zheng, Xiaojiao , Tianlu Chen , Aihua Zhao , Zhangchi Ning , Junliang Kuang , Shouli Wang , Yijun You , et al. 2021. “Hyocholic Acid Species as Novel Biomarkers for Metabolic Disorders.” Nature Communications 12: 1487. 10.1038/s41467-021-21744-w PMC7935989 33674561

[11] Zhong, Jing , Xiaofang He , Xinxin Gao , Qiaohong Liu , Yu Zhao , Ying Hong , Weize Zhu , et al. 2023. “Hyodeoxycholic Acid Ameliorates Nonalcoholic Fatty Liver Disease by Inhibiting Ran‐Mediated Pparα Nucleus‐Cytoplasm Shuttling.” Nature Communications 14: 5451. 10.1038/s41467-023-41061-8 PMC10482907 37673856

[12] Kuang, Junliang , Jieyi Wang , Yitao Li , Mengci Li , Mingliang Zhao , Kun Ge , and Dan Zheng , et al. 2023. “Hyodeoxycholic Acid Alleviates Non‐Alcoholic Fatty Liver Disease Through Modulating the Gut‐Liver Axis.” Cell Metabolism 35: 1752–1766.e8. 10.1016/j.cmet.2023.07.011 37591244

[13] Sánchez, Borja . 2018. “Bile Acid–microbiota Crosstalk in Gastrointestinal Inflammation and Carcinogenesis: A Role for Bifidobacteria and Lactobacilli?” Nature Reviews Gastroenterology & Hepatology 15: 205205. 10.1038/nrgastro.2018.23 29512648

[14] Gehrig, Jeanette L. , Siddarth Venkatesh , Hao‐Wei Chang , Matthew C. Hibberd , Vanderlene L. Kung , Jiye Cheng , Robert Y. Chen , et al. 2019. “Effects of Microbiota‐directed Foods in Gnotobiotic Animals and Undernourished Children.” Science 365: eaau4732. 10.1126/science.aau4732 31296738 PMC6683325

[15] Gonzalez, Liara M. , Adam J. Moeser , and Anthony T. Blikslager . 2015. “Porcine Models of Digestive Disease: The Future of Large Animal Translational Research.” Translational Research 166: 12–27. 10.1016/j.trsl.2015.01.004 25655839 PMC4458388

[16] Ziegler, Amanda , Liara Gonzalez , and Anthony Blikslager . 2016. “Large Animal Models: The Key to Translational Discovery in Digestive Disease Research.” Cellular and Molecular Gastroenterology and Hepatology 2: 716–724. 10.1016/j.jcmgh.2016.09.003 28090566 PMC5235339

[17] Le Chatelier, Emmanuelle , Trine Nielsen , Junjie Qin , Edi Prifti , Falk Hildebrand , Gwen Falony , Mathieu Almeida , et al. 2013. “Richness of Human Gut Microbiome Correlates With Metabolic Markers.” Nature 500: 541–546. 10.1038/nature12506 23985870

[18] Turnbaugh, Peter J. , Micah Hamady , Tanya Yatsunenko , Brandi L. Cantarel , Alexis Duncan , Ruth E. Ley , Mitchell L. Sogin , et al. 2009. “Core Gut Microbiome in Obese and Lean Twins.” Nature 457: 480–484. 10.1038/nature07540 19043404 PMC2677729

[19] Yang, Hua , Yun Xiang , Kelsy Robinson , Junjun Wang , Guolong Zhang , Jiangchao Zhao , and Yingping Xiao . 2018. “Gut Microbiota Is a Major Contributor to Adiposity in Pigs.” Frontiers in Microbiology 9: 03045. 10.3389/fmicb.2018.03045 PMC6296290 30619136

[20] Pang, Xiaoyan , Xiuguo Hua , Qian Yang , Dezhong Ding , Chuanyan Che , Li Cui , Wei Jia , Peter Bucheli , and Liping Zhao . 2007. “Inter‐Species Transplantation of Gut Microbiota From Human to Pigs.” The ISME Journal 1: 156–162. 10.1038/ismej.2007.23 18043625

[21] Diao, H. , H. L. Yan , Y. Xiao , B. Yu , J. Yu , J. He , P. Zheng , et al. 2016. “Intestinal Microbiota Could Transfer Host Gut Characteristics From Pigs to Mice.” BMC Microbiology 16: 238. 10.1186/s12866-016-0851-z 27729007 PMC5057279

[22] Mauricio, María D , Eva Serna , María Leonor Fernández‐Murga , Jesica Portero , Martín Aldasoro , Soraya L. Valles , Yolanda Sanz , and José M. Vila . 2017. “ *Bifidobacterium Pseudocatenulatum* CECT 7765 Supplementation Restores Altered Vascular Function in An Experimental Model of Obese Mice.” International Journal of Medical Sciences 14: 444–451. 10.7150/ijms.18354 28539820 PMC5441036

[23] Moya‐Pérez, Angela , Alexander Neef , and Yolanda Sanz . 2015. “ *Bifidobacterium Pseudocatenulatum* CECT 7765 Reduces Obesity‐Associated Inflammation by Restoring the Lymphocyte‐Macrophage Balance and Gut Microbiota Structure in High‐Fat Diet‐Fed Mice.” PLoS One 10: e0126976. 10.1371/journal.pone.0126976 26161548 PMC4498624

[24] Jia, Wei , Meilin Wei , Cynthia Rajani , and Xiaojiao Zheng . 2021. “Targeting the Alternative Bile Acid Synthetic Pathway for Metabolic Diseases.” Protein & Cell 12: 411–425. 10.1007/s13238-020-00804-9 33252713 PMC8106556

[25] Zhao, Qing , Huan Ren , Nian Yang , Xuyang Xia , Qifeng Chen , Dingding Zhou , Zhaoqian Liu , et al. 2023. “ *Bifidobacterium Pseudocatenulatum*‐Mediated Bile Acid Metabolism to Prevent Rheumatoid Arthritis via the Gut–Joint Axis.” Nutrients 15: 255. 10.3390/nu15020255. https://www.mdpi.com/2072-6643/15/2/255 36678126 PMC9861548

[26] Jarocki, Piotr , Marcin Podleśny , Paweł Glibowski , and Zdzisław Targoński . 2014. “A New Insight Into the Physiological Role of Bile Salt Hydrolase Among Intestinal Bacteria From the Genus Bifidobacterium.” PLoS One 9: e114379. 10.1371/journal.pone.0114379 25470405 PMC4255033

[27] Adhikari, Arijit A. , Tom C. M. Seegar , Scott B. Ficarro , Megan D. McCurry , Deepti Ramachandran , Lina Yao , Snehal N. Chaudhari , et al. 2020. “Development of a Covalent Inhibitor of Gut Bacterial Bile Salt Hydrolases.” Nature Chemical Biology 16: 318–326. 10.1038/s41589-020-0467-3 32042200 PMC7036035

[28] Lynch, Jonathan B. , Erika L. Gonzalez , Kayli Choy , Kym F. Faull , Talia Jewell , Abelardo Arellano , Jennifer Liang , et al. 2023. “Gut Microbiota Turicibacter Strains Differentially Modify Bile Acids and Host Lipids.” Nature Communications 14: 3669. 10.1038/s41467-023-39403-7 PMC10281990 37339963

[29] Joyce, Susan A. , John MacSharry , Patrick G. Casey , Michael Kinsella , Eileen F. Murphy , Fergus Shanahan , Colin Hill , and Cormac G. M. Gahan . 2014. “Regulation of Host Weight Gain and Lipid Metabolism by Bacterial Bile Acid Modification in the Gut.” Proceedings of the National Academy of Sciences 111: 7421–7426. 10.1073/pnas.1323599111 PMC4034235 24799697

[30] Li, Fei , Changtao Jiang , Kristopher W. Krausz , Yunfei Li , Istvan Albert , Haiping Hao , Kristin M. Fabre , et al. 2013. “Microbiome Remodelling Leads to Inhibition of Intestinal Farnesoid X Receptor Signalling and Decreased Obesity.” Nature Communications 4: 2384. 10.1038/ncomms3384 PMC6595219 24064762

[31] Li, Xiao , Jie Yang , Xiaofeng Zhou , Chen Dai , Mengmeng Kong , Linshan Xie , Chenglin Liu , et al. 2024. “Ketogenic Diet‐induced Bile Acids Protect Against Obesity Through Reduced Calorie Absorption.” Nature Metabolism 6: 1397–1414. 10.1038/s42255-024-01072-1 38937659

[32] Zhong, Xian‐chun , Ya‐meng Liu , Xiao‐Xia Gao , Kristopher W. Krausz , Bing Niu , Frank J. Gonzalez , and Cen Xie . 2023. “Caffeic Acid Phenethyl Ester Suppresses Intestinal FXR Signaling and Ameliorates Nonalcoholic Fatty Liver Disease by Inhibiting Bacterial Bile Salt Hydrolase Activity.” Acta Pharmacologica Sinica 44: 145–156. 10.1038/s41401-022-00921-7 35655096 PMC9813015

[33] Wei, Meilin , Fengjie Huang , Ling Zhao , Yunjing Zhang , Wei Yang , Shouli Wang , Mengci Li , et al. 2020. “A Dysregulated Bile Acid‐Gut Microbiota Axis Contributes to Obesity Susceptibility.” EBioMedicine 55: 102766. 10.1016/j.ebiom.2020.102766 32408110 PMC7225614

[34] Chaudhari, Snehal N. , David A. Harris , Hassan Aliakbarian , James N. Luo , Matthew T. Henke , Renuka Subramaniam , Ashley H. Vernon , et al. 2021. “Bariatric Surgery Reveals a Gut‐Restricted Tgr5 Agonist With Anti‐Diabetic Effects.” Nature Chemical Biology 17: 20–29. 10.1038/s41589-020-0604-z 32747812 PMC7891870

[35] Chen, Yingjia , Snehal N. Chaudhari , David A. Harris , Cullen F. Roberts , Andrei Moscalu , Vasundhara Mathur , Lei Zhao , et al. 2024. “A Small Intestinal Bile Acid Modulates the Gut Microbiome to Improve Host Metabolic Phenotypes Following Bariatric Surgery.” Cell Host & Microbe 32: 1315–1330. 10.1016/j.chom.2024.06.014 39043190 PMC11332993

[36] Hu, Jun , Libao Ma , Yangfan Nie , Jianwei Chen , Wenyong Zheng , Xinkai Wang , Chunlin Xie , et al 2018. “A Microbiota‐Derived Bacteriocin Targets the Host to Confer Diarrhea Resistance in Early‐Weaned Piglets.” Cell Host & Microbe 24: 817–832. 10.1016/j.chom.2018.11.006 30543777

[37] Hu, Jun , Jianwei Chen , Xiaojian Xu , Qiliang Hou , Jing Ren , and Xianghua Yan . 2023. “Gut Microbiota‐Derived 3‐Phenylpropionic Acid Promotes Intestinal Epithelial Barrier Function Via AhR Signaling.” Microbiome 11: 102. 10.1186/s40168-023-01551-9 37158970 PMC10165798

[38] Fluhr, Leviel , Uria Mor , Aleksandra A. Kolodziejczyk , Mally Dori‐Bachash , Avner Leshem , Shlomik Itav , Yotam Cohen , et al. 2021. “Gut Microbiota Modulates Weight Gain in Mice After Discontinued Smoke Exposure.” Nature 600: 713–719. 10.1038/s41586-021-04194-8 34880502

[39] Wong, Sunny H. , Liuyang Zhao , Xiang Zhang , Geicho Nakatsu , Juqiang Han , Weiqi Xu , and Xue Xiao , et al. 2017. “Gavage of Fecal Samples From Patients With Colorectal Cancer Promotes Intestinal Carcinogenesis in Germ‐Free and Conventional Mice.” Gastroenterology 153: 1621–1633.e6. 10.1053/j.gastro.2017.08.022 28823860

[40] Zeng, Xiangjun , Xiaoqing Li , Xia Li , Cong Wei , Ce Shi , Kejia Hu , Delin Kong , et al. 2023. “Fecal Microbiota Transplantation From Young Mice Rejuvenates Aged Hematopoietic Stem Cells by Suppressing Inflammation.” Blood 141: 1691–1707. 10.1182/blood.2022017514 36638348 PMC10646769

[41] Cano, Paola Gauffin , Arlette Santacruz , Fernando M. Trejo , and Yolanda Sanz . 2013. “Bifidobacterium CECT 7765 Improves Metabolic and Immunological Alterations Associated With Obesity in High‐Fat Diet‐Fed Mice.” Obesity 21: 2310–2321. 10.1002/oby.20330 23418126

[42] Sato, Yuko , Koji Atarashi , Damian R. Plichta , Yasumichi Arai , Satoshi Sasajima , Sean M. Kearney , Wataru Suda , et al. 2021. “Novel Bile Acid Biosynthetic Pathways are Enriched in the Microbiome of Centenarians.” Nature 599: 458–464. 10.1038/s41586-021-03832-5 34325466

[43] Magoč, Tanja , and Steven L. Salzberg . 2011. “FLASH: Fast Length Adjustment of Short Reads to Improve Genome Assemblies.” Bioinformatics 27: 2957–2963. 10.1093/bioinformatics/btr507 21903629 PMC3198573

[44] Bolger, Anthony M. , Marc Lohse , and Bjoern Usadel . 2014. “Trimmomatic: A Flexible Trimmer for Illumina Sequence Data.” Bioinformatics 30: 2114–2120. 10.1093/bioinformatics/btu170 24695404 PMC4103590

[45] Edgar, Robert C. , Brian J. Haas , Jose C. Clemente , Christopher Quince , and Rob Knight . 2011. “UCHIME Improves Sensitivity and Speed of Chimera Detection.” Bioinformatics 27: 2194–2200. 10.1093/bioinformatics/btr381 21700674 PMC3150044

[46] McDonald, Daniel , Morgan N. Price , Julia Goodrich , Eric P. Nawrocki , Todd Z. DeSantis , Alexander Probst , Gary L. Andersen , Rob Knight , and Philip Hugenholtz . 2012. “An Improved Greengenes Taxonomy With Explicit Ranks for Ecological and Evolutionary Analyses of Bacteria and Archaea.” The ISME Journal 6: 610–618. 10.1038/ismej.2011.139 22134646 PMC3280142

[47] Stubbs, Thomas M. , Marc Jan Bonder , Anne‐Katrien Stark , Felix Krueger , Ferdinand von Meyenn , Oliver Stegle , Wolf Reik , et al. 2017. “Multi‐Tissue DNA Methylation Age Predictor in Mouse.” Genome Biology 18: 68. 10.1186/s13059-017-1203-5 28399939 PMC5389178

[48] Lee, Phil Hyoun , and Hagit Shatkay . 2006. “BNTagger: Improved Tagging SNP Selection Using Bayesian Networks.” Bioinformatics 22: e211–e219. 10.1093/bioinformatics/btl233 16873474

[49] Hyatt, Doug , Gwo‐Liang Chen , Philip F. LoCascio , Miriam L. Land , Frank W. Larimer , and Loren J. Hauser . 2010. “Prodigal: Prokaryotic Gene Recognition and Translation Initiation Site Identification.” BMC Bioinformatics 11: 119. 10.1186/1471-2105-11-119 20211023 PMC2848648

[50] Zhang, Hao , Jiawan Wang , Jianghua Shen , Siqi Chen , Hailong Yuan , Xuan Zhang , Xu Liu , et al. 2024. “Prophylactic Supplementation With Bifidobacterium Infantis or Its Metabolite Inosine Attenuates Cardiac Ischemia/Reperfusion Injury.” iMeta 3: e220. 10.1002/imt2.220 39135700 PMC11316933

[51] Liu, Hongbin , Qingqing Lv , and Lei Dai . 2020. “Quantitative Analysis of 16S rRNA Gene Copies in Mouse Fecal Sample.” In Microbiome Protocols eBook, Bio-101, e2003368. 10.21769/BioProtoc.2003368

[52] Matsuki, Takahiro , Koichi Watanabe , Ryuichiro Tanaka , and Hiroshi Oyaizu . 1998. “Rapid Identification of Human Intestinal Bifidobacteria by 16s rRNA‐Targeted Species‐ and Group‐Specific Primers.” FEMS Microbiology Letters 167: 113–121. 10.1111/j.1574-6968.1998.tb13216.x 9809413

[53] Wang, Jing , Liming Zeng , Bie Tan , Guangran Li , Bo Huang , Xia Xiong , Fengna Li , et al. 2016. “Developmental Changes in Intercellular Junctions and Kv Channels in the Intestine of Piglets During the Suckling and Post‐Weaning Periods.” Journal of Animal Science and Biotechnology 7: 4. 10.1186/s40104-016-0063-2 26819706 PMC4729073

[54] Brown, Gregory Thomas , and David E. Kleiner . 2016. “Histopathology of Nonalcoholic Fatty Liver Disease and Nonalcoholic Steatohepatitis.” Metabolism 65: 1080–1086. 10.1016/j.metabol.2015.11.008 26775559 PMC4889547

[55] Le Roy, Tiphaine , Marta Llopis , Patricia Lepage , Aurélia Bruneau , Sylvie Rabot , Claudia Bevilacqua , Patrice Martin , et al. 2013. “Intestinal Microbiota Determines Development of Non‐alcoholic Fatty Liver Disease In Mice.” Gut 62: 1787–1794. 10.1136/gutjnl-2012-303816 23197411

[56] Hernandez, Gabriella V. , Victoria A. Smith , Megan Melnyk , Matthew A. Burd , Kimberly A. Sprayberry , Mark S. Edwards , Daniel G. Peterson , et al. 2020. “Dysregulated FXR‐FGF19 Signaling and Choline Metabolism Are Associated With Gut Dysbiosis and Hyperplasia in a Novel Pig Model of Pediatric NASH.” American Journal of Physiology—Gastrointestinal and Liver Physiology 318: G582–G609. 10.1152/ajpgi.00344.2019 32003601 PMC7099491

[57] Yang, Tingting , Ting Shu , Guanlan Liu , Huifang Mei , Xiaoyu Zhu , Xin Huang , Luyong Zhang , and Zhenzhou Jiang . 2017. “Quantitative Profiling ōf 19 Bile Acids in Rat Plasma, Liver, Bile and Different Intestinal Section Contents to Investigate Bile Acid Homeostasis and the Application of Temporal Variation of Endogenous Bile Acids.” The Journal of Steroid Biochemistry and Molecular Biology 172: 69–78. 10.1016/j.jsbmb.2017.05.015 28583875

[58] Zhang, Chaozheng , Yu Zheng , Shenxi Ma , and Zhiguo Wu . 2017. “Determination of Bile Acids in Rat Cecal Contents by LC–MS.” Chromatographia 80: 1733–1739. 10.1007/s10337-017-3395-y

[59] Qi, Ming , Simeng Liao , Jing Wang , Yuankun Deng , Andong Zha , Yirui Shao , Zhijuan Cui , et al. 2022. “MyD88 Deficiency Ameliorates Weight Loss Caused by Intestinal Oxidative Injury in an Autophagy‐Dependent Mechanism.” Journal of Cachexia, Sarcopenia and Muscle 13: 677–695. 10.1002/jcsm.12858 34811946 PMC8818611

