# Supplementary material for: Gut Bifidobacterium pseudocatenulatum protects against fat deposition by enhancing secondary bile acid biosynthesis
Source: Imeta. 2024 Dec 30;3(6):e261. doi: 10.1002/imt2.261 (PMC11683477; doi:10.1002/imt2.261)
Supplement: Supplementary file 1 — Figure S1. Comparison of lipid metabolism pathways in the colonic microbiota. Figure S2. The impact of the colonic microbiome on hepatic fat deposition varies among different breed. Figure S3. Identification of differential bacterial biomarkers between Duroc × Landrace × Yorkshire and Ningxiang pigs by linear discriminant analysis effect size. Figure S4. Colonic microbiome mediates variations in secondary bile acid metabolism between lean and obese pigs. Figure S5. Bifidobacterium pseudocatenulatum attenuates hepatic steatosis in high‐fat diet‐fed mice. Figure S6. Bifidobacterium pseudocatenulatum attenuates fat deposition in antibiotic‐pretreated high‐fat diet fed mice. Figure S7. GR7 impairs Bifidobacterium pseudocatenulatum attenuates hepatic steatosis in high‐fat diet ‐fed mice. Figure S8. Lithocholic acid attenuates hepatic steatosis in high‐fat diet‐fed rats and Ningxiang pigs. [file IMT2-3-e261-s002.docx]

**Supporting information to**

**Gut** ***Bifidobacterium pseudocatenulatum* protects against fat deposition by enhancing secondary bile acid biosynthesis**

**Running title:** *Bifidobacterium pseudocatenulatum* protects against fat deposition

Andong Zha^1,2,3,4^, Ming Qi^4^, Yuankun Deng^1,2^, Hao Li^1,2^, Nan Wang^1,2^, Chengming Wang^1,2^, Simeng Liao^1,2^, Dan Wan^4^, Xia Xiong^4^, Peng Liao^4^, Jing Wang^1,2*^, Yulong Yin ^1,2,4*^, Bi’e Tan^1,2*^

^1^Key Laboratory of Hunan Province for the Products Quality Regulation of Livestock and Poultry, College of Animal Science and Technology, Hunan Agricultural University, Changsha 410128, China.

^2^Yuelushan Laboratory, Hunan 410128, China.

^3^School of Basic Medical Science, Central South University, Changsha 410001, China

^4^Laboratory of Animal Nutritional Physiology and Metabolic Process, Key Laboratory of Agro-ecological Processes in Subtropical Region, National Engineering Laboratory for Pollution Control and Waste Utilization in Livestock and Poultry Production, Institute of Subtropical Agriculture, Chinese Academy of Sciences, Changsha 410125, China.

*Correspondence: [jingwang023@hunau.edu.cn](mailto:jingwang023@hunau.edu.cn) (Jing Wang), [yinyulong@isa.ac.cn](mailto:yinyulong@isa.ac.cn) (Yulong Yin), and [bietan@hunau.edu.cn](mailto:bietan@hunau.edu.cn) (Bi’e Tan).

**Supplementary figures**


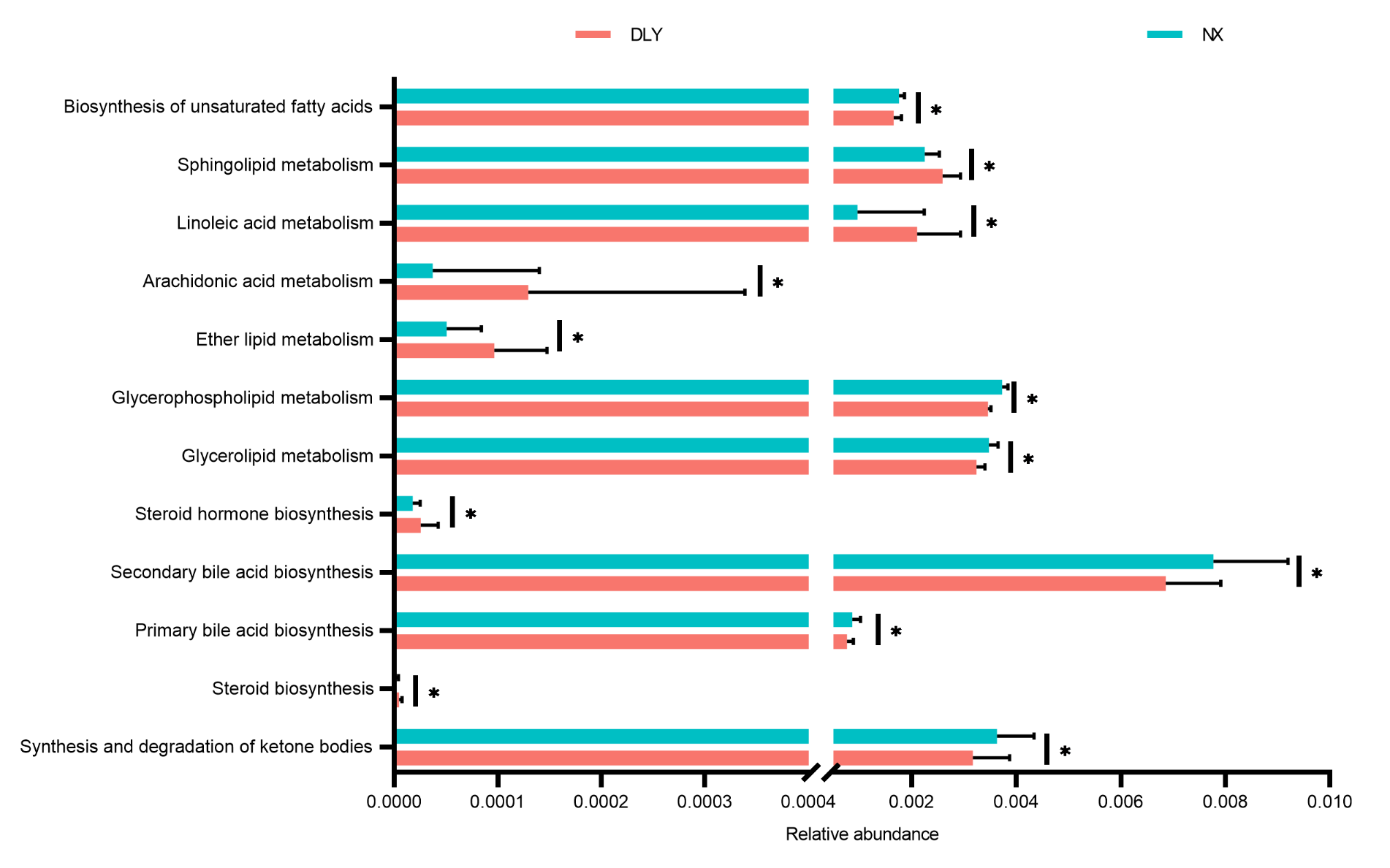


**Figure S1 Comparison of lipid metabolism pathways in the colonic microbiota.** Data are shown as mean ± SEMs, *n* = 40 per group. **p* < 0.05 (Student’s *t* test). DLY group, Duroc × Landrace × Yorkshire pigs; NX group, Ningxiang pigs.

**Figure S2 The impact of the colonic microbiome on hepatic fat deposition varies among different breed.** (A) Serum biochemical profiling in chow diet fed mice; DLY-CD: Mice received colonic microbiome from Duroc × Landrace × Yorkshire (DLY) pigs and fed with chow diet; NX-CD: Mice received colonic microbiome from Ningxiang (NX) pigs and fed with chow diet. Low-density lipoprotein cholesterol (LDLC), high-density lipoprotein cholesterol (HDLC), non-esterified fatty acid (NEFA). Data are shown as mean ± SEMs, *n* = 8 per group. **p* < 0.05 (Student’s *t* test). (B) Liver weight. Data are shown as the mean ± SEMs, *n* = 8 per group. **p* < 0.05 (Student’s *t* test). (C) Representative H&E staining of liver, and the hepatic steatosis score. Data are shown as mean ± SEMs, *n* = 4 per group. **p* < 0.05 (Student’s *t* test), scale bar = 50 μm. (D) The mRNA expression of genes related to lipogenesis in liver in colonic microbiota transplantation experiment. Data are shown as mean ± SEMs, *n* = 8 per group. **p* < 0.05 (Student’s *t* test). Peroxisome proliferator-activated receptor gamma coactivator 1-alpha (*Pgc1α*), Hormone-sensitive lipase (*Hsl*), Carnitine palmitoyltransferase 1 beta (*Cpt1β*), Peroxisome proliferator-activated receptor alpha (*Ppaα*), Acetyl-CoA carboxylase (*Acc*), Farnesoid X receptor (*Fxr*), Fatty acid synthase (*Fasn*), Takeda G protein-coupled receptor 5 (*Tgr5*), Peroxisome proliferator-activated receptor gamma (*Pparγ*), Sterol regulatory element-binding protein 1 (*Srebp1*), Lipoprotein lipase (*Lpl*)*.* (E) Serum biochemical profiling in high fat diet fed mice; DLY-HFD: Mice received colonic microbiome from DLY and fed with high fat diet; NX-HFD: Mice received colonic microbiome from DLY and fed with high fat diet. low-density lipoprotein cholesterol (LDLC), high-density lipoprotein cholesterol (HDLC), non-esterified fatty acid (NEFA). Data are shown as mean ± SEMs, *n* = 8 per group. **p* < 0.05 (Student’s *t* test). (F) Liver weight. Data are shown as mean ± SEMs, *n* = 8 per group. **P* < 0.05 (Student’s *t* test). (G) Representative H&E staining of liver and hepatic steatosis score. Data are shown as mean ± SEMs, *n* = 4 per group. **p* < 0.05 (Student’s *t* test), scale bar = 50 μm. (H) The mRNA expression of genes related to lipogenesis in liver in colonic microbiota transplantation experiment. Data are shown as mean ± SEMs, *n* = 8 per group. **p* < 0.05 (Student’s *t* test). Peroxisome proliferator-activated receptor gamma coactivator 1-alpha (*Pgc1α*), Hormone-sensitive lipase (*Hsl*), Carnitine palmitoyltransferase 1 beta (*Cpt1β*), Peroxisome proliferator-activated receptor alpha (*Ppaα*), Acetyl-CoA carboxylase (*Acc*), Farnesoid X receptor (*Fxr*), Fatty acid synthase (*Fasn*), Takeda G protein-coupled receptor 5 (*Tgr5*), Peroxisome proliferator-activated receptor gamma (*Pparγ*), Sterol regulatory element-binding protein 1 (*Srebp1*), Lipoprotein lipase (*Lpl*)*.* DLY-CD: Mice received colonic microbiome from DLY pigs and fed with chow diet; NX-CD: Mice received colonic microbiome from NX pigs and fed with chow diet; DLY-HFD: Mice received colonic microbiome from DLY pigs and fed with HFD; NX-CD: Mice received colonic microbiome from NX pigs and fed with HFD.


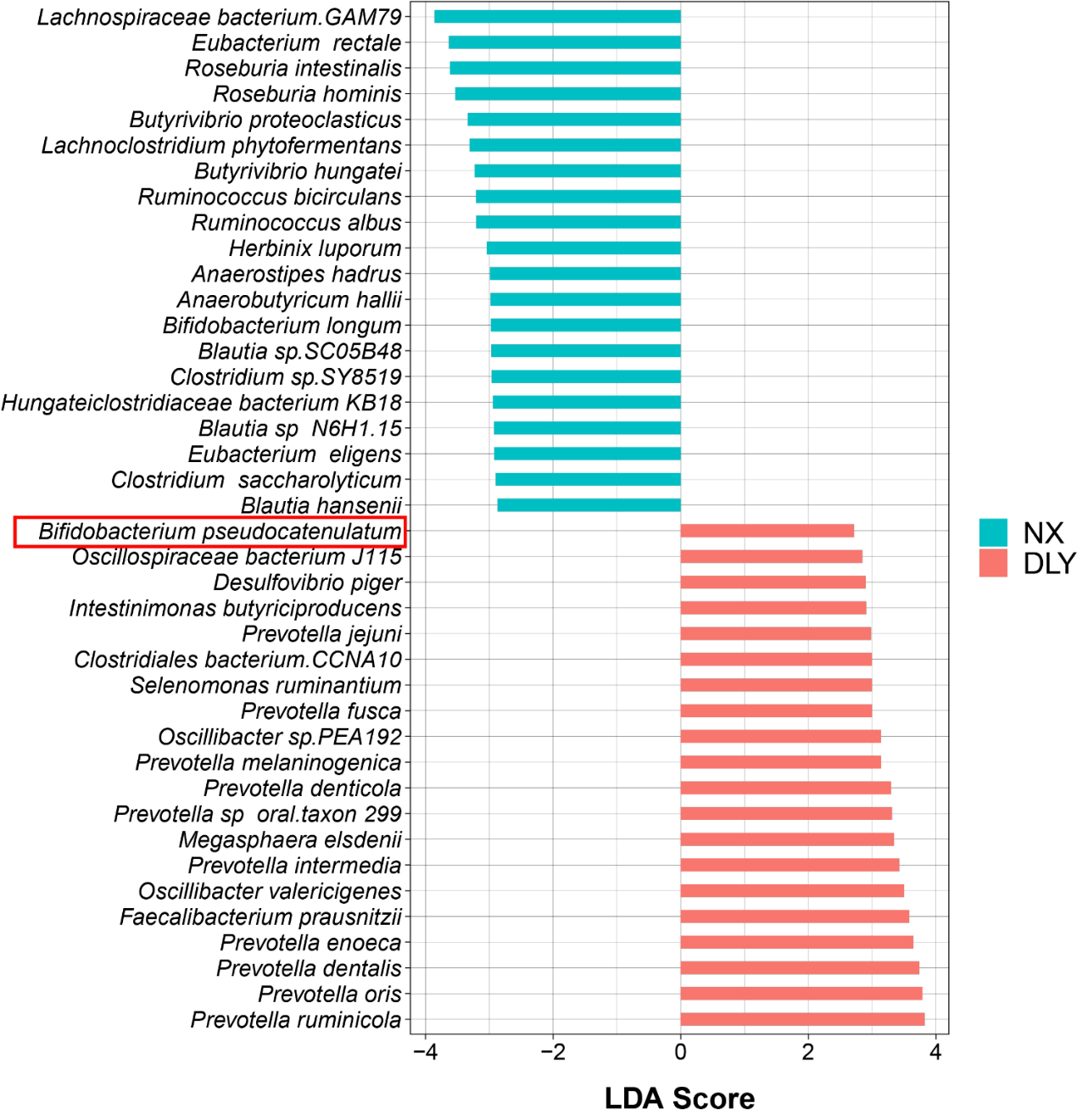


**Figure S3 Identification of differential bacterial biomarkers between Duroc × Landrace × Yorkshire and Ningxiang pigs by linear discriminant analysis effect size.** Only the top 20 species are shown. Linear discriminant analysis (LDA), DLY group, Duroc × Landrace × Yorkshire pigs; NX group, Ningxiang pigs.

**Figure S4 Colonic microbiome mediates variations in secondary bile acid metabolism between lean and obese pigs.** (A) Linear discriminant analysis (LDA) score representing the KEGG pathways (level 3) with significant differences between Duroc × Landrace × Yorkshire (DLY) and Ningxiang (NX) pigs. Only top 10 pathways are shown. (B) The percentage of bile acid species in the colon of DLY and NX pigs. Data are shown as the mean ± SEMs, *n* = 8 per group. **p* < 0.05 (Student’s *t* test). Deoxycholic acid (DCA), Chenodeoxycholic acid (CDCA), Muricholic acid (MCA), Lithocholic acid (LCA), Hyocholic acid (HCA), Hyodeoxycholic acid (HDCA), Ursodeoxycholic acid (UDCA). (C) The differential metabolites in secondary bile acids metabolism among DLY and NX pigs. Data are shown as the mean ± SEMs, *n* = 8 per group. **p* < 0.05 (Student’s *t* test). Glycochenodeoxycholic acid (GCDCA), Taurochenodeoxycholic acid (TCDCA), Chenodeoxycholic acid (CDCA), Lithocholic acid (LCA), Iso-Lithocholic acid (iso LCA), Glycolithocholic acid (GLCA), Hyodeoxycholic acid (HDCA), Hyocholic acid (HCA), β-Muricholic acid (β MCA). (D) Two-tailed Spearman correlation coefficient analysis of differential bile acids and backfat thickness (*p* < 0.05). (E) Two-tailed Spearman correlation coefficient analysis of gut microbiota and bile acids. + means a positive correlation; － means a negative correlation. (*p* < 0.05). Glycohyocholic acid (GHCA), Chenodeoxycholic acid (CDCA), Glycochenodeoxycholic acid (GCDCA), Hyocholic acid (HCA), Taurochenodeoxycholic acid (TCDCA), Taurohyodeoxycholic acid (THDCA), 7-Dehydrocholic acid (7-HDCA), 7-Ketolithocholic acid (7-Keto LCA), Glycohyodeoxycholic acid (GHDCA), β-Muricholic acid (β MCA), Glycolithocholic acid (GLCA), Iso-lithocholic acid (iso LCA), Lithocholic acid (LCA), Hyodeoxycholic acid (HDCA), α-Muricholic acid (α MCA), Deoxycholic acid (DCA), ω Muricholic acid (ωMCA), Ursodeoxycholic acid (UDCA), *β*-Ursodeoxycholic acid, Nor-Cholic acid (NorCA). DLY group, Duroc × Landrace × Yorkshire pigs; NX group, Ningxiang pigs.


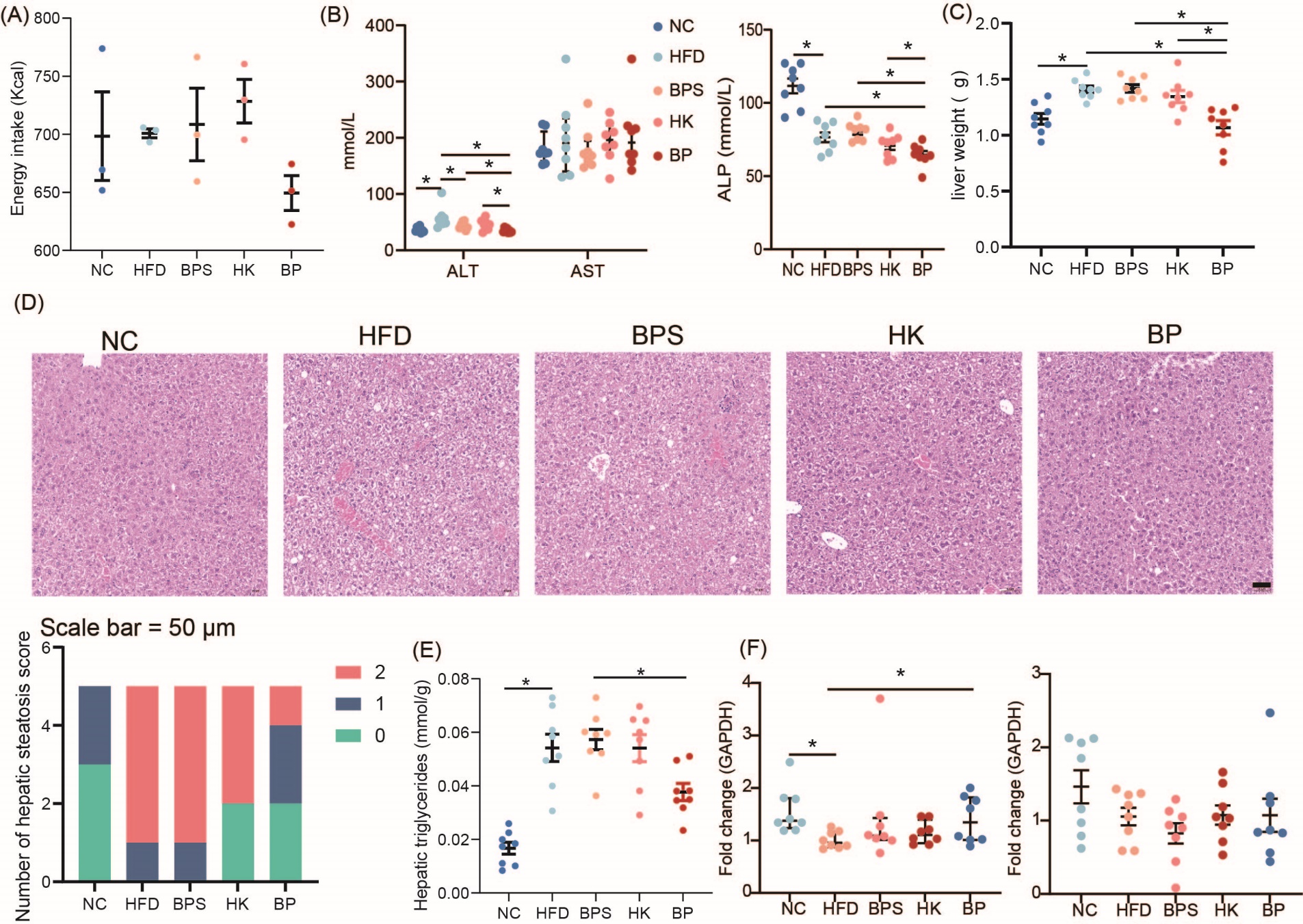


**Figure S5 *B. pseudocatenulatum* attenuates hepatic steatosis in high fat diet-fed mice.** (A) Accumulative energy intake. Data are shown as mean ± SEMs, *n* = 3 per group. **p* < 0.05 (kruskal-wallis test). (B) Serum alanine aminotransferase (ALT) (median with interquartile range, kruskal-wallis test), aspartate aminotransferase (AST) (median with interquartile range, kruskal-wallis test), and alkaline phosphatase (ALP) (mean ± SEMs, one-way ANOVA) concentrations. *n* = 8 per group. **p* < 0.05 (). (C) Liver weight. Data are shown as mean ± SEMs, *n* = 8 per group. **p* < 0.05 (one-way ANOVA). (D) Representative H&E-stained image in liver, and hepatic steatosis score, scale bar = 50 μm. (E) Hepatic triglyceride concentration. Data are shown as mean ± SEMs, *n* = 8 per group. **p* < 0.05 (one-way ANOVA). (F) The mRNA expression of *Fxr* (median with interquartile range, kruskal-wallis test) and *Tgr5* (mean ± SEMs, one-way ANOVA) in liver. *n* = 8 per group. **p* < 0.05. Farnesoid X receptor (*Fxr*), Takeda G protein-coupled receptor 5 (*Tgr5*). NC group: chow diet + vehicle; HFD group: HFD + vehicle; BPS group: HFD + *B. pseudocatenulatum* supernatant; HK group: HFD + heat-killed *B. pseudocatenulatum*; BP group: HFD + live *B. pseudocatenulatum*.


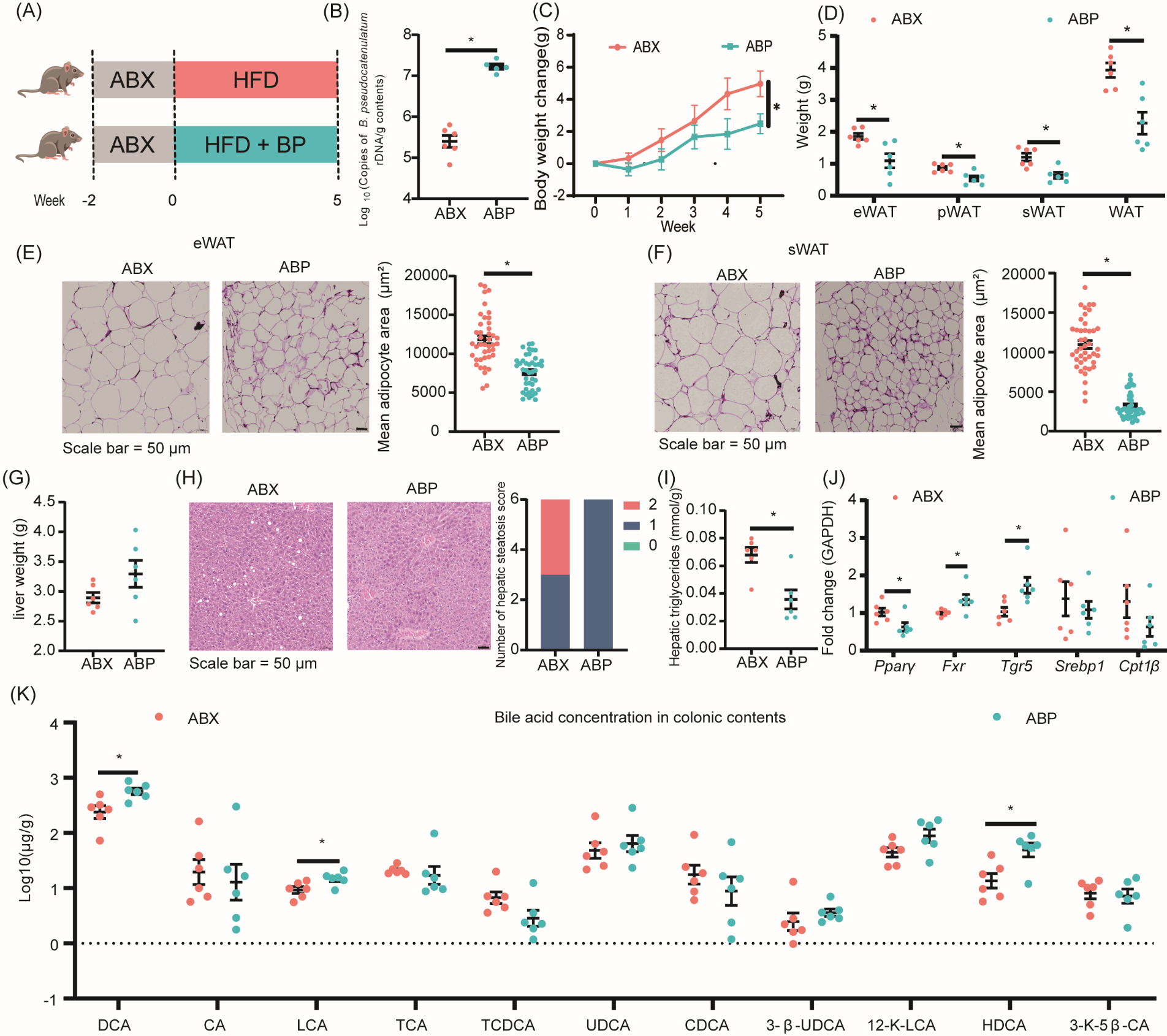
**Figure S6 *B. pseudocatenulatum* attenuates fat deposition in antibiotic-pretreated high fat diet fed mice. (A)** Schematic of the *B. pseudocatenulatum* monocolonization experiment in antibiotic-pretreated HFD-fed mice. C57BL/6 male mice were pretreated with antibiotic cocktail for 2 weeks, and gavage with *B. pseudocatenulatum* for 5 weeks. Mice were fed with HFD during experimental period. ABX group: antibiotic treatment + HFD + BBL medium; ABP group: antibiotic treatment + HFD + live *B. pseudocatenulatum.* (B) The copies of *B. pseudocatenulatum* 16S rDNA in colonic contents. Data are shown as mean ± SEMs, *n* = 6 per group. **p* < 0.05 (Student’s *t* test). (C) Body weight change. Data are shown as mean ± SEMs, *n* = 6 per group. **p* < 0.05 (repeated measures ANOVA). (D) Adipose tissue weight. epididymal white adipose tissue (eWAT), perirenal white adipose tissue (pWAT), subcutaneous white adipose tissue (sWAT), white adipose tissue (WAT). Data are shown as mean ± SEMs, *n* = 6 per group. **p* < 0.05 (Student’s *t* test). (E) Mean adipocyte area, and representative H&E-stained image of eWAT. Data are shown as mean ± SEMs, n = *6* per group. **p* < 0.05 (Student’s *t* test). (F) Mean adipocyte area, and representative H&E-stained image of iWAT. Data are shown as mean ± SEMs, *n* = 8 per group. **p* < 0.05 (Student’s *t* test). (G) Liver weight. Data are shown as mean ± SEMs, *n* = 6 per group. **p* < 0.05 (Student’s *t* test). (H) Representative H&E-stained image in liver, and hepatic steatosis score. Data are shown as mean ± SEMs, *n* = 6 per group. **p* < 0.05 (Student’s *t* test), scale bar = 50 μm. (I) Hepatic triglyceride concentration. Data are shown as mean ± SEMs, *n* = 6 per group. **p* < 0.05 (Student’s *t* test). (J) The mRNA expression of lipid metabolism-related genes. Data are shown as mean ± SEMs, *n* = 6 per group. **p* < 0.05 (Student’s *t* test). Peroxisome proliferator-activated receptor gamma (*Pparγ*), Farnesoid X receptor (*Fxr*), Takeda G protein-coupled receptor 5 (*Tgr5*), Sterol regulatory element-binding protein 1 (*Srebp1*), Carnitine palmitoyltransferase 1 beta (*Cpt1β*). (K) Bile acid concentration in colonic contents. Data are shown as mean ± SEMs, *n* = 6 per group. **p* < 0.05 (Student’s *t* test). ABX group: antibiotic treatment + HFD + BBL medium; ABP group: antibiotic treatment + HFD + live *B. pseudocatenulatum.*


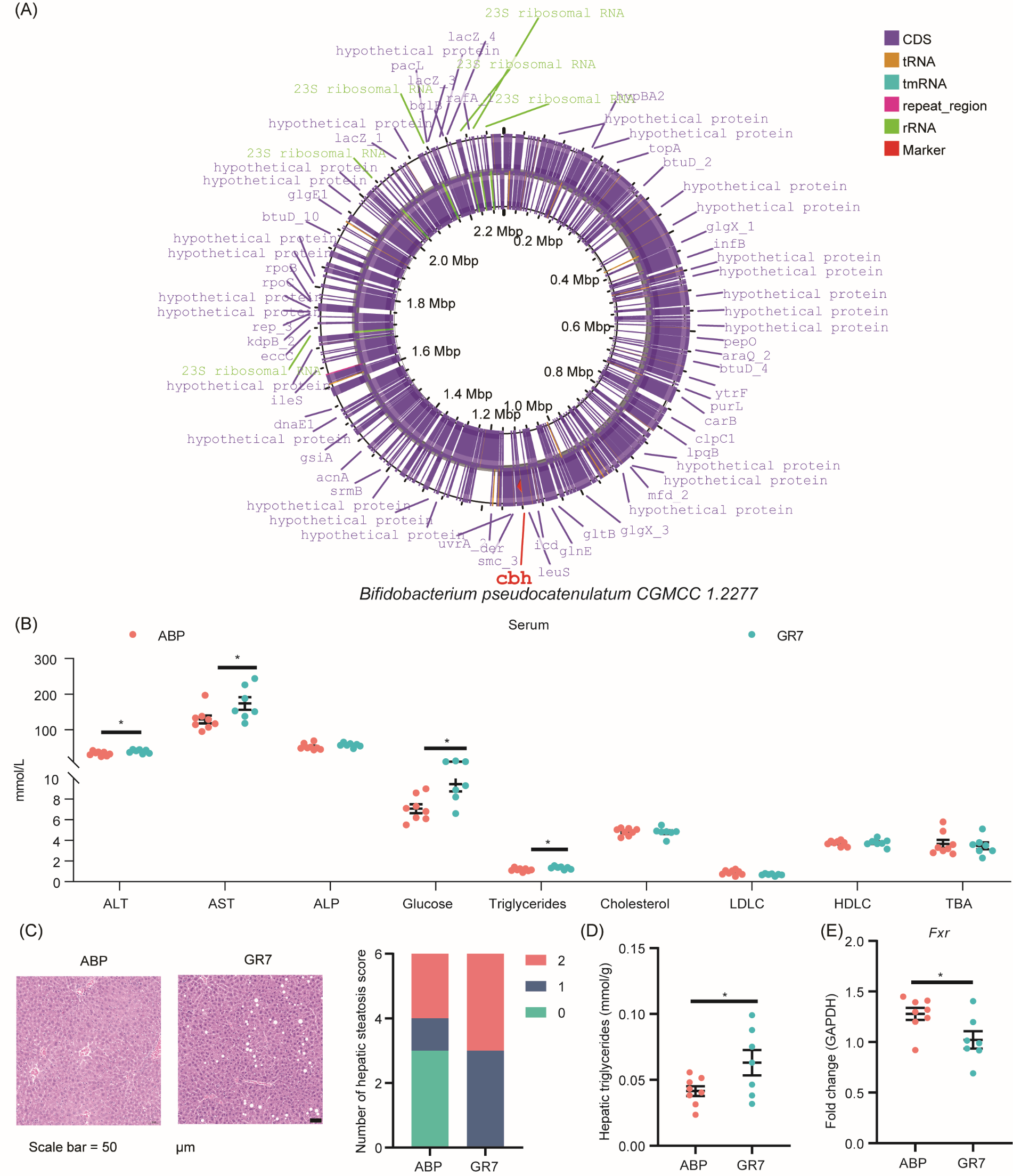
**Figure S7 GR7 impairs *B. pseudocatenulatum* attenuates hepatic steatosis in high fat diet -fed mice.** (A) Functional prediction of the *B. pseudocatenulatum* genome. (B) Serum biochemical profiling in high fat diet (HFD)-fed mice. alanine aminotransferase (ALT), aspartate aminotransferase (AST), alkaline phosphatase (ALP), low-density lipoprotein cholesterol (LDLC), high-density lipoprotein cholesterol (HDLC). ABP group: antibiotic treatment + HFD + live *B. pseudocatenulatum*; GR7 group: antibiotic treatment + HFD + live *B. pseudocatenulatum* + GR7. Data are shown as the mean ± SEMs, ABP (*n* = 8), GR7 (*n* = 7). **p* < 0.05 (Student’s *t* test). (C) Representative H&E-stained image in liver, and hepatic steatosis score. Data are shown as mean ± SEMs, *n* = 6 per group. **p* < 0.05 (Student’s *t* test), scale bar = 50 μm. (D) Hepatic triglyceride concentrations. Data are shown as mean ± SEMs, ABP (*n* = 8), GR7(*n* = 7). **p* < 0.05 (Student’s *t* test). (E) The mRNA expression of *Fxr* in liver. Data are shown as mean ± SEMs, ABP (*n* = 8), GR7(*n* = 7). **p* < 0.05 (Student’s *t* test). Farnesoid X receptor (*Fxr*). ABP group: HFD + ABX + live *B. pseudocatenulatum +* Vehicle; GR7 group: HFD + ABX + live *B. pseudocatenulatum* + GR7.


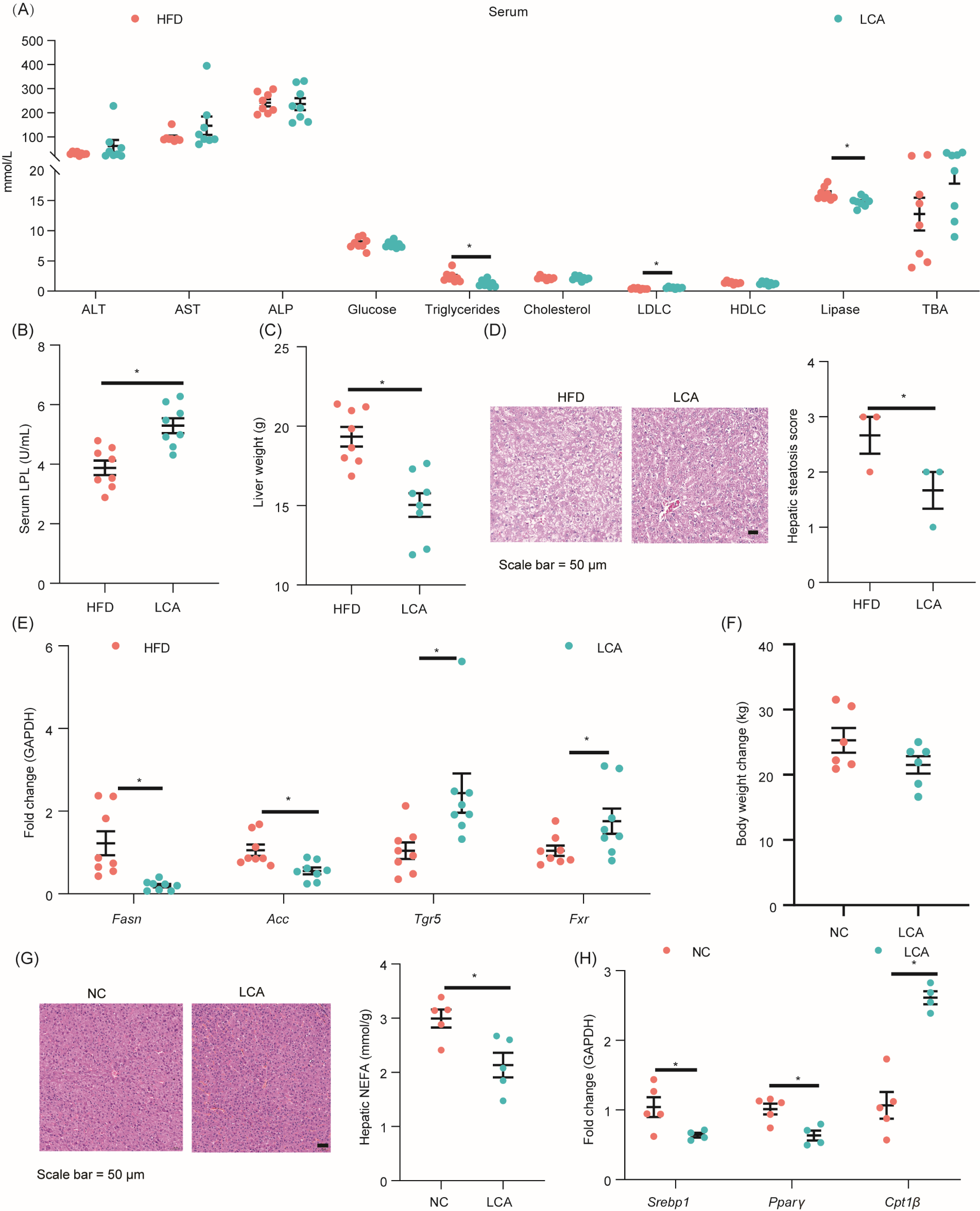


**Figure S8 LCA attenuates hepatic steatosis in high fat diet-fed rats and Ningxiang pigs.** (A) Serum biochemical profiling in high fat diet (HFD)-fed rat. Alanine aminotransferase (ALT), aspartate aminotransferase (AST), alkaline phosphatase (ALP), low-density lipoprotein cholesterol (LDLC), high-density lipoprotein cholesterol (HDLC), lipase (LPL), non-esterified fatty acid (NEFA), total bile acids (TBA). HFD group: HFD-fed rat; LCA: 0.3% lithocholic acid supplemented HFD-fed rat. Data are shown as mean ± SEMs, *n* = 8 per group. **p* < 0.05 (Student’s *t* test). (B) Serum lipoprotein lipase (LPL) activity. Data are shown as mean ± SEMs, *n* = 8 per group, * *p <* 0.05 (student's *t* test). HFD group: HFD-fed rat; LCA: 0.3% lithocholic acid supplemented HFD-fed rat. Data are shown as mean ± SEMs, *n* = 8 per group. **p* < 0.05 (Student’s *t* test). (C) Liver weight. HFD group: HFD-fed rat; LCA: 0.3% lithocholic acid supplemented HFD-fed rat. Data are shown as mean ± SEMs, *n* = 8 per group. **p* < 0.05 (Student’s *t* test). (D) Representative H&E-stained image in liver, and hepatic steatosis score. HFD group: HFD-fed rat; LCA: 0.3% lithocholic acid supplemented HFD-fed rat. Data are shown as mean ± SEMs, *n* = 8 per group. **p* < 0.05 (Student’s *t* test), scale bar = 50 μm. (E) The mRNA expression of lipid metabolism related genes in liver. HFD group: HFD-fed rat; LCA: 0.3% lithocholic acid supplemented HFD-fed rat. Data are shown as mean ± SEMs, *n* = 8 per group. **p* < 0.05 (Student’s *t* test). Fatty acid synthase (*Fasn*), Acetyl-coA carboxylase (*Acc*), Takeda G protein-coupled receptor 5 (*Tgr5*), Farnesoid X receptor (*Fxr*). (F) Body weight change. Data are shown as mean ± SEMs, *n* = 6 per group, **p* < 0.05 (student's *t* test). NC group: pigs fed a basal diet; LCA group: pigs fed a basal diet supplemented with 792 mg/kg lithocholic acid. (G) Representative H&E-stained image in liver, and hepatic non-esterified fatty acid (NEFA) concentration. NC group: basal diet fed NX pigs, LCA group: 792 mg/kg lithocholic acid supplemented basal diet fed NX pigs. Data are shown as mean ± SEMs, *n* = 5 per group. **p* < 0.05 (Student’s *t* test), scale bar = 50 μm. (H) The mRNA expression of lipid metabolism related genes in liver. NC group: basal diet fed NX pigs, LCA group: 792 mg/kg lithocholic acid supplemented basal diet fed NX pigs. Data are shown as mean ± SEMs, *n* = 5 per group. **p* < 0.05 (Student’s *t* test). Sterol regulatory element-binding protein 1 (*Srebp1*), Peroxisome proliferator-activated receptor gamma (*Pparγ*), Carnitine palmitoyltransferase 1 beta (*Cpt1β*).
